# Dynamic interplay of maternal and paternal contributions to offspring phenotype in Eurasian perch

**DOI:** 10.1186/s12915-026-02602-x

**Published:** 2026-04-20

**Authors:** Rossella Debernardis, Abhipsa Panda, Sylwia Wałdowska, Katarzyna Palińska-Żarska, Christophe Klopp, Taina Rocha de Almeida, Sylwia Jarmołowicz, Piotr Hliwa, Daniel Żarski

**Affiliations:** 1 Team of Reproduction and Development in Fish, InLife Institute of Animal Reproduction and Food Research, Polish Academy of Sciences, Olsztyn, Poland; 2 Department of Ichthyology, Hydrobiology and Aquatic Ecology, National Inland Fisheries Research Institute, Oczapowskiego 10, 10-719, Olsztyn, Poland; 3 Université de Toulouse, GenoToul Bioinformatic Facility, Sigenae, BioinfOmics, UR875 MIAT, INRAE, Castanet‐Tolosan, France; 4 Department of Ichthyology and Aquaculture, University of Warmia and Mazury in Olsztyn, Oczapowskiego 5, 10-719 Olsztyn, Poland

**Keywords:** Transcriptomics, Fish larvae, Development, Maternal-effect genes, Paternal-effect genes

## Abstract

**Background:**

Parental contributions to offspring phenotype extend beyond genetic inheritance, encompassing non-genetic factors that influence early development. However, the interplay between maternal and paternal effects remains poorly understood. This study investigates these contributions in Eurasian perch (*Perca fluviatilis*) — a valuable model to study parental effect in finfishes — by analyzing early life traits and transcriptomic profiles of larvae resulting from crosses between wild and domesticated parents.

**Results:**

Maternal effects dominated key developmental traits, including hatching success, growth, and swim bladder inflation. Transcriptomic analysis revealed a complex regulatory interplay, with 573 genes under exclusive maternal control, while no genes were solely influenced by paternal input. Maternal-effect genes were primarily associated with metabolic, developmental, and stress-response pathways, shaping early larval physiology. Further analysis identified Eurasian-perch-specific candidate maternal-effect genes, such as *crtac1*, *slc16a7*, *cox5b*, *kdr*, *cald1*, and *bin2*, highlighting their potential role in early development. Although paternal contributions were limited, a subset of genes exhibited conditional paternal influence, suggesting a nuanced parental interplay in gene expression regulation.

**Conclusions:**

These findings challenge the traditional perspective of strictly coordinated parental contributions, instead revealing a dynamic parental interplay over gene expression and offspring traits. The dominance of maternal effects suggests a primary role in shaping early development, while paternal factors may modulate expression patterns in a context-dependent manner. This study enhances our understanding of parental effects in fish, providing valuable insights for aquaculture breeding strategies and evolutionary biology research.

**Supplementary Information:**

The online version contains supplementary material available at 10.1186/s12915-026-02602-x.

## Background

Early development is shaped by the combined influences of both mother and father, collectively referred to as parental effect [1, 2]. These influences may originate from both genetic and non-genetic parental contributions but are frequently mediated by mechanisms independent of DNA sequence variation [3, 4]. There is a growing body of evidence that parental contribution extends to gamete derived non-genetic inheritance (NGI) factors — such as mRNAs, small RNAs, proteins, metabolites, and epigenetic modifications — which can reflect environmental conditions and parental experiences [5]. Alongside offspring’s genotype, NGI factors constitute a significant component of parental investment and collectively are pivotal in shaping progeny traits, impacting on development, survival, and evolutionary success from early life stages [6–9].

Within this broader framework, maternal effects are defined as the influences of the mothers on offspring phenotype that occur beyond the direct effects on the offspring’s genotype [10]. These effects are transmitted through oocytes and can arise from the mother’s genetic makeup or be influenced by her experiences and environment. For example, maternal experiences (such as diet, stress or physiological conditions) can alter the molecular composition of the eggs, including lipids [11, 12], proteins [13], mRNAs [14], and non-coding RNAs [15, 16], thereby influencing offspring development [17, 18]. Maternal effects have historically received the greatest attention and are often considered the primary drivers of offspring traits [8, 19]; however, they represent only part of the parental influence on offspring.

Growing evidence shows that fathers also play significant roles in offspring development beyond only transmission of alleles [4]. Importantly, a substantial component of these paternal influences is attributed to non-genetic mechanisms, frequently mediated by sperm-derived factors such as sperm-derived epigenetic marks, small RNAs, and environmentally induced modifications [9, 20, 21]. Offspring phenotype thus reflects the combined action of genetic inheritance and non-genetic parental effects from both parents. Despite this, research continues to focus on maternal and paternal influences separately rather than in an integrated framework. In contrast, disentangling these maternal and paternal contributions is essential for understanding how larval phenotypes are formed, providing valuable insights for targeted breeding strategies and evolutionary studies.

Parental effects have been studied across various taxa [1], yet fish are particularly suitable for investigating the complexities of parents’ interactions due to their high fecundity and developmental plasticity [22]. Also, the majority of fishes exhibit external fertilization [23] allowing straightforward monitoring of the development from the moment of fertilization. Up to now, studies have primarily focused on investigating how certain parental traits in fish (i.e., length, weight, colour, egg size and content) affect offspring development and performance [7, 24, 25]. On the contrary, very few investigations have explored the impact of parental effects on progeny transcriptome [26, 27], and even fewer have examined the potential correlation between larvae’s zootechnical traits and molecular profiles, in relation to the influence of parental contributions [28]. Despite some progress, many gaps in knowledge still remain. One of these lies in the complex interactions between mothers and fathers [19], whether through cooperation or context-dependent contributions that ultimately determine the fate of their offspring. In fact, the understandings of the roleplay of maternal and paternal effects to offspring, as well as quantifying each parent’s specific impact on the offspring’s gene expression profile, remain poorly understood [25]. This presents a valuable opportunity for further exploration into how both maternal and paternal contributions shape the phenotype of future generations, thereby contributing to the broader understanding of evolutionary processes.

Parental contributions include direct impact on offspring gene expression during development [8], adding a further layer of complexity to the offspring’s underlying phenotype. In this context, transcriptomics is a valuable tool for examining how parental effects influence gene expression and, consequently, progeny phenotype [28, 29]. Therefore, by leveraging transcriptome analysis, it is possible to gain deeper insights into how parents play their role in affecting offspring development and performance.

One way to investigate parental effect is by crossing individuals originating from two extreme phenotypes, yielding viable progeny [6, 19]. In the case of fish, several studies have highlighted considerable phenotypic differences between domesticated and wild fish, which can emerge very early in the domestication process, even in the first generation, as a result of significant epigenetic modifications [30]. These variations can alter traits such as growth and immunity, affecting organism’s performance and fitness [31]. In Eurasian perch, available data indicate that domestication impacts reproduction [32], alters the molecular composition of eggs [33], and affects larval traits such as digestion and immune function from the earliest stages of development [34, 35]. Taking all of this into account, reciprocal crosses between domesticated and wild populations, whose phenotypes differ markedly, provide a powerful framework for disentangling and quantifying parental effects at the progeny level. This pronounced phenotypic contrast makes Eurasian perch an ideal model for investigating the specific maternal and paternal contributions examined in this study.

The present study adopts a more combined approach to explore how both parents interact to shape offspring phenotype. Therefore, we created unique paired reciprocal crosses between wild and domesticated Eurasian perch (*Perca fluviatilis*) spawners to separately analyze maternal and paternal contributions to offspring phenotype and gene expression. Importantly, our objective was not to examine domestication effects per se, but rather to use these groups as biologically distinct parental backgrounds. This allowed us to examine which zootechnical traits and transcriptomic consequences in the offspring are controlled by either mother or father. A subset of freshly hatched larvae from each family was used for whole-organism transcriptome profiling at the mouth-opening stage, while the remaining larvae were reared separately under controlled conditions to evaluate growth and survival during the larval period. With this design we aimed at identifying maternal- and paternal-effect traits and genes, offering novel insights into the complexity of early developmental inheritance. By disentangling these effects, our study contributes to a deeper understanding of transgenerational trait transmission in fishes, with potential implications for both aquaculture breeding strategies and evolutionary biology.

## Results

### Zootechnical data

**Fig. 1:**
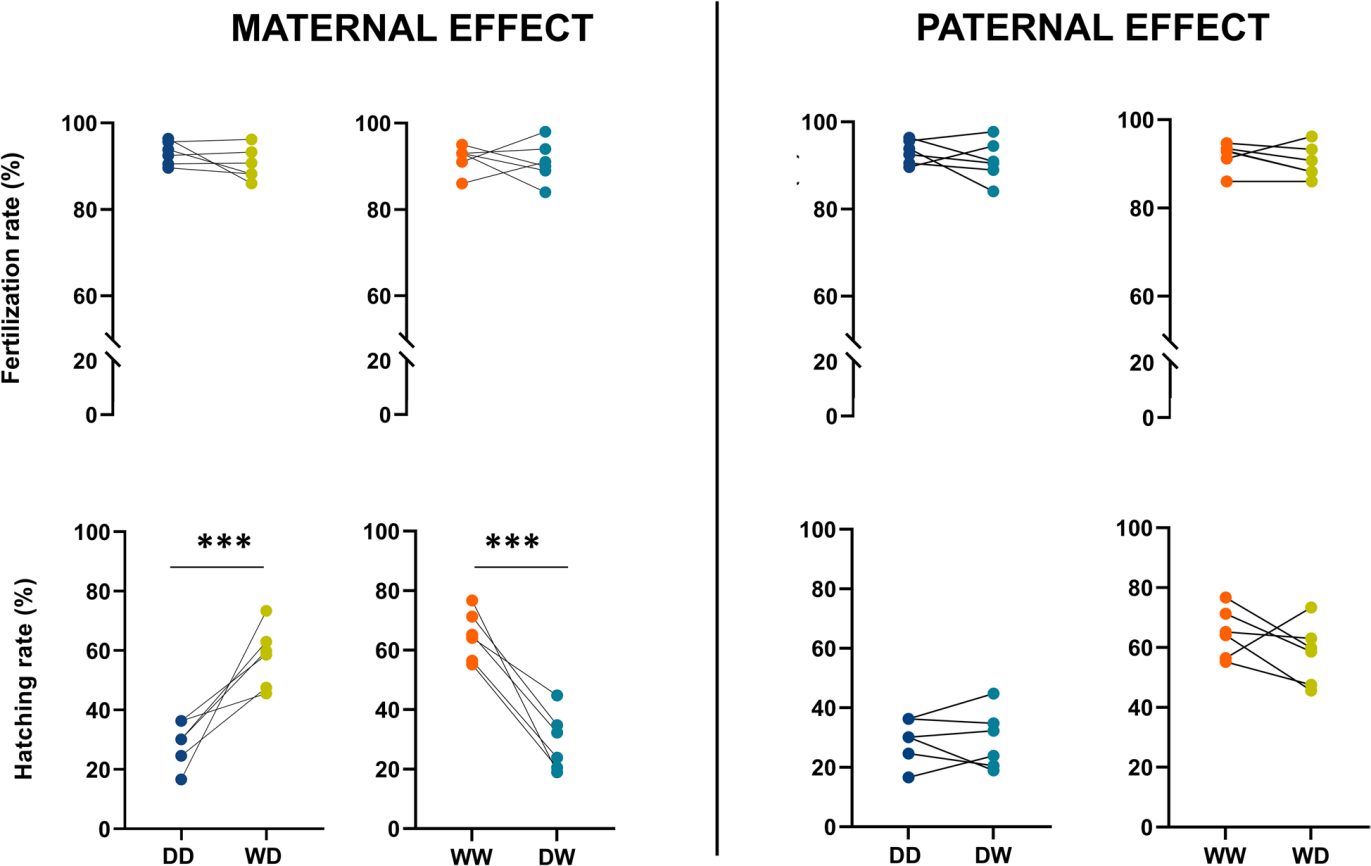
Fertilization rate and hatching rate for all the families of Eurasian perch. The asterisks (**p < 0.01, ***p < 0.001, ****p < 0.0001) show significant differences between the groups. Additional statistical information is provided in Additional File 6

No significant differences in fertilization rates were observed when comparing the influence of both paternal- and maternal-effects across groups (Fig. 1). However, when analysing hatching rates, significant differences were only observed in the maternal-effect analysis, with wild females consistently exhibited the highest hatching rates, regardless of the male’s origin (Fig. 1). Also, no differences were observed for deformity rate (in Additional file 1: Fig. S1). For Type 1 cannibalism (where the prey is not fully digested), the only significant difference emerged for maternal effect analysis, specifically while comparing groups where females were paired with wild (W) males (in Additional file 1: Fig. S1).

**Fig. 2:**
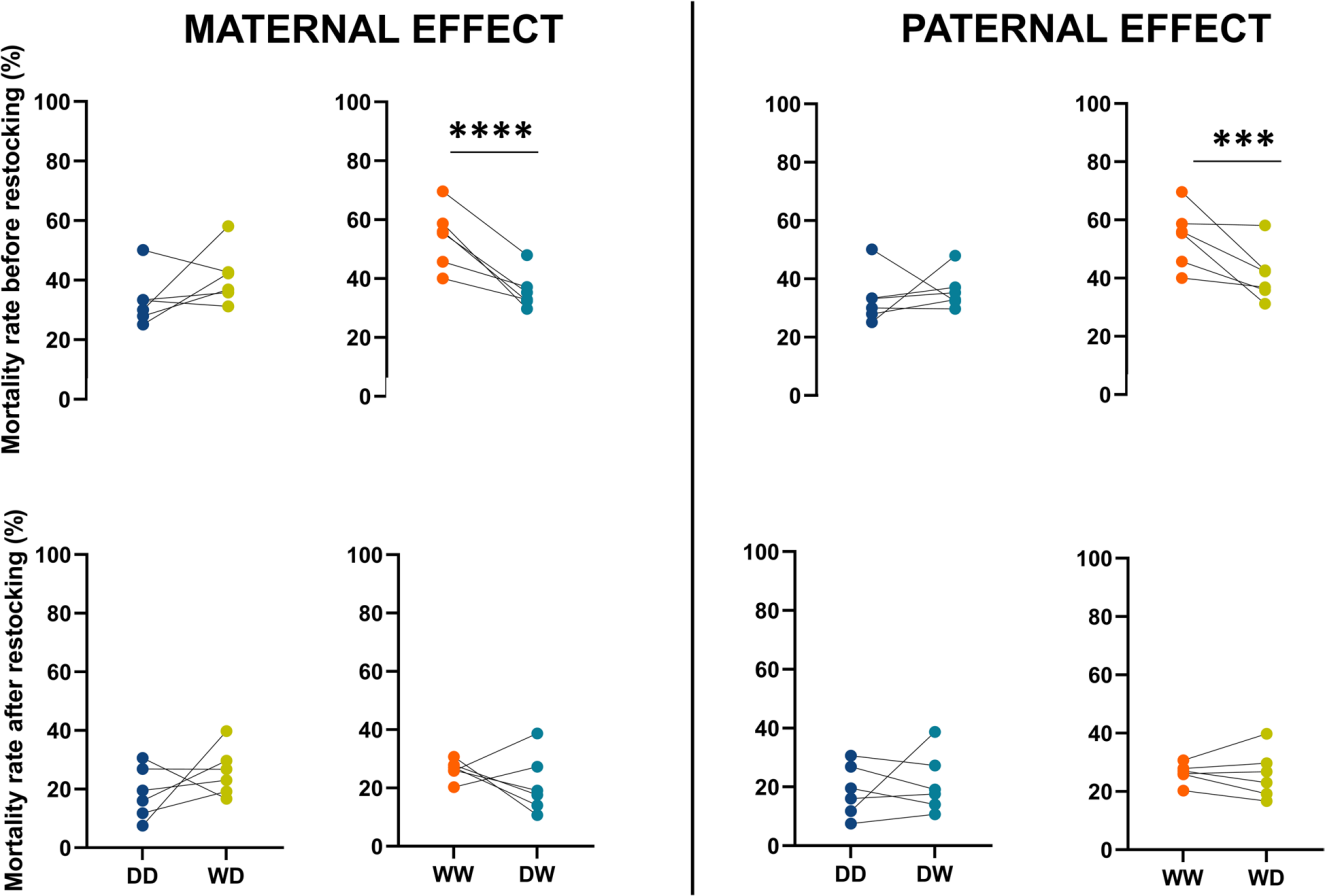
Mortality rate before and after restocking for all the families of Eurasian perch. The asterisks (***p < 0.001, ****p < 0.0001) show significant differences between the groups. Additional statistical information is provided in Additional File 6

A significant difference in mortality rates before weaning was observed when groups were compared as WW (wild♀ × wild♂) vs DW (dom♀ × wild♂) (for maternal effect) and WW vs WD (wild♀ × dom♂) (for paternal effect), with the WW group always showing a significantly higher mortality rate (Fig. 2). Indeed, mortality rates were higher in larvae with a predominantly wild phenotype compared to those originating from domesticated parents as shown also in the cumulative mortality graphs (in Additional file 1: Fig. S2-S5).

**Fig. 3:**
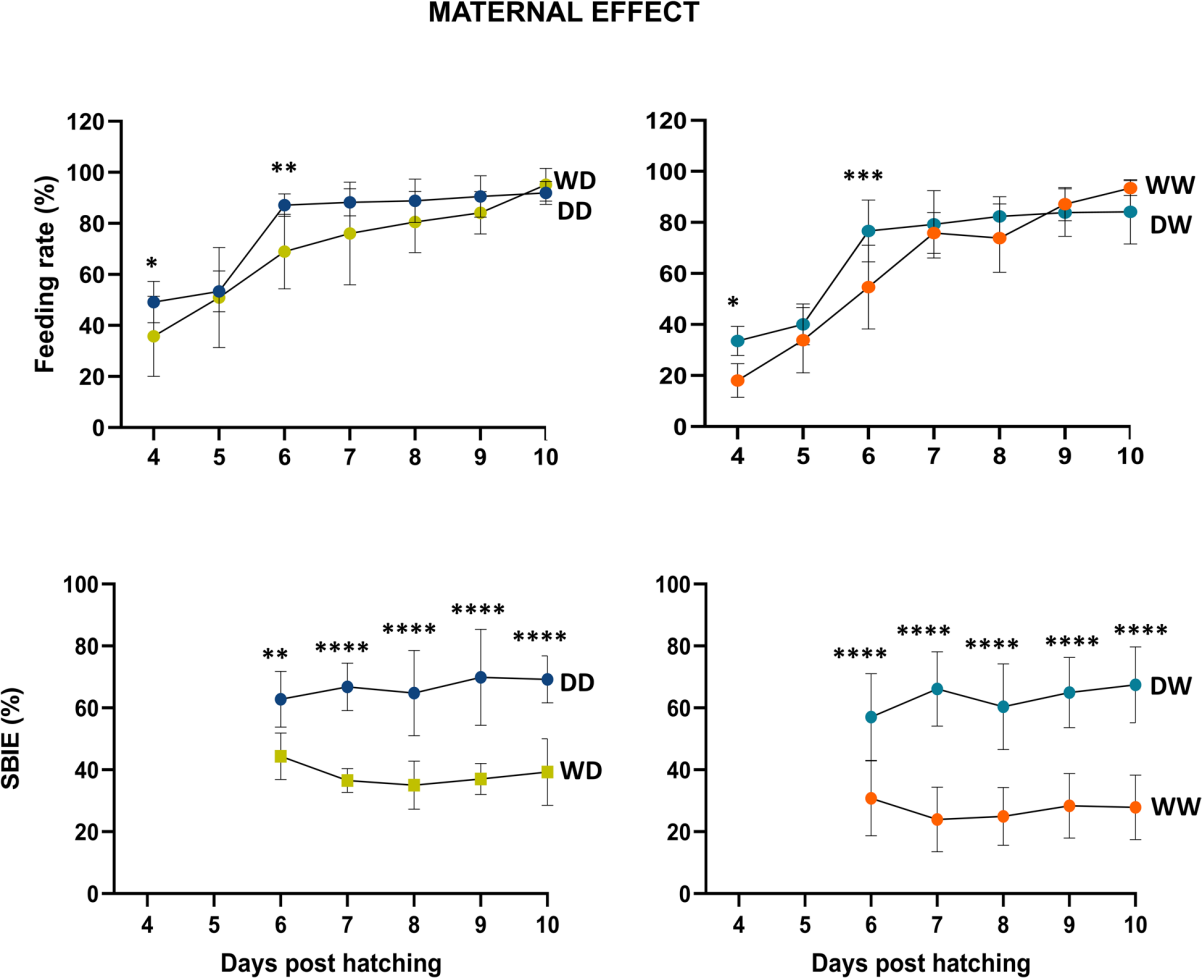
Feeding rate and SBIE kinetics (%) for all Eurasian perch families analysed for maternal-effect. The asterisks (*p < 0.05, **p < 0.01, ***p < 0.001) show significant differences between the groups over time. SBIE — swim bladder inflation effectiveness. Error bars indicate standard deviation (SD). Additional statistical information is provided in Additional File 6

**Fig. 4:**
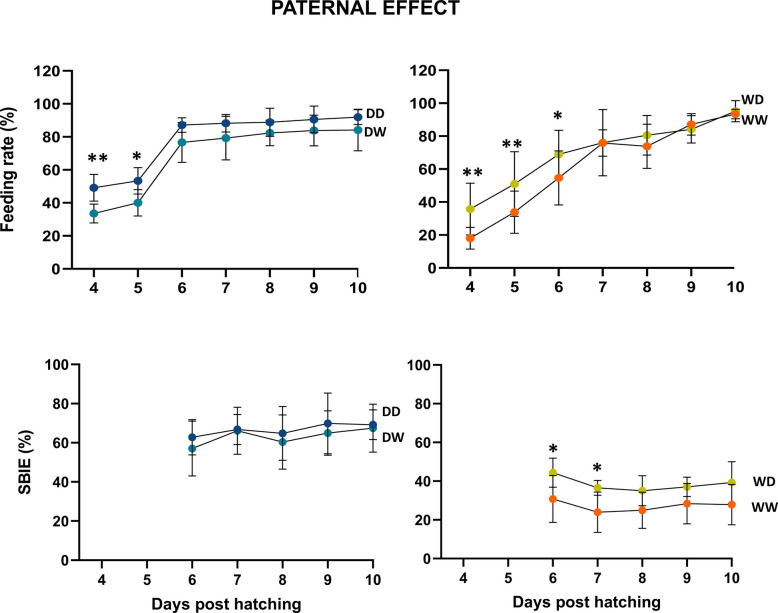
Feeding rate and SBIE kinetics (%) for all Eurasian perch families analysed for paternal-effect. The asterisks (*p < 0.05) show significant differences between the groups over time. Error bars indicate standard deviation (SD). Additional statistical information is provided in Additional File 6

The feeding rate was evaluated over a 7-day period (from 4 to 10 days post hatching — DPH) to study kinetics of feeding onset (Figs. 3 and 4). Significant differences were mainly observed when data were analysed for paternal effect (Fig. 4). No significant differences were detected in the yolk sac measurements among groups (in Additional file 1: Fig. S1). In contrast, the analysis of swim bladder inflation effectiveness (SBIE) over a 5-day period (from 6 to 10 DPH) revealed significant daily differences tied to maternal influences (Fig. 3). Moreover, the larvae coming from domesticated (D) females consistently showed a higher SBIE, regardless of the phenotype of the male they were paired with.

**Fig. 5:**
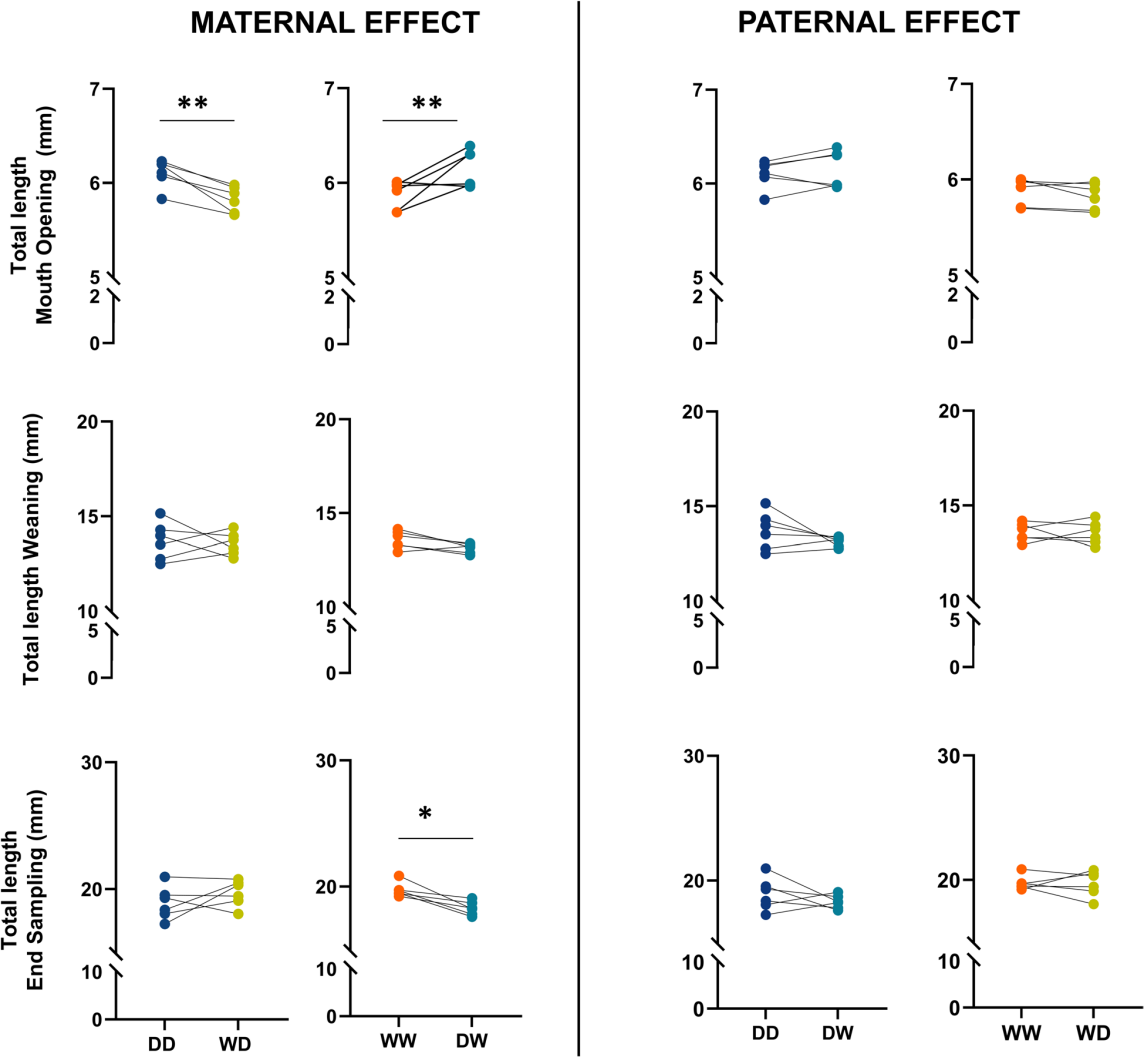
Total length (mm) of larvae measured at different developmental stages for all Eurasian perch families. The asterisks (*p < 0.05, **p < 0.01) show significant differences between the groups over time. Additional statistical information is provided in Additional File 6

Significant differences in total length (TL) (Fig. 5) were observed across the groups both at the mouth-opening stage and at the end of the larval period (referred to as end sampling in the graphs), exclusively in relation to the maternal-effect analyses. No significant differences were observed between groups analysed for paternal-effect.

**Fig. 6:**
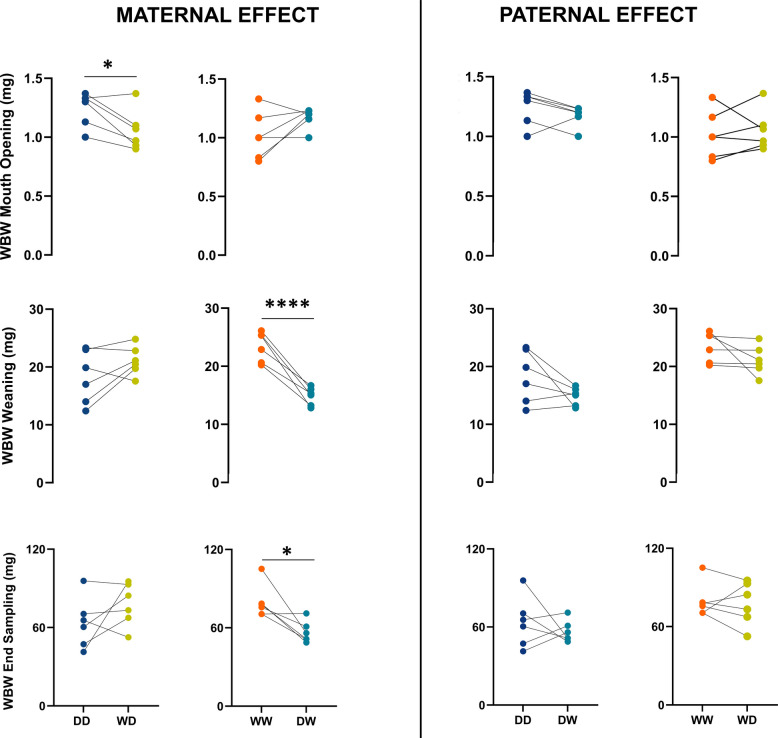
Wet body weight (WBW) (mg) of larvae measured at different developmental stages for all Eurasian perch families. The asterisks (*p < 0.05, ****p < 0.0001) show significant differences between the groups over time. Additional statistical information is provided in Additional File 6

For wet body weight (WBW) (Fig. 6), most significant differences were observed in the maternal-effect analysis. Although larvae from D females were characterized by higher WBW at the mouth-opening stage, by weaning and at the end of the experiment, larvae from W females exhibited higher WBW. Regarding paternal-effects, a significant difference was only observed at weaning, specifically when comparing the WW and WD groups.

In summary, zootechnical data analysis revealed that females seem to play a dominant role compared to males, across several parameter related to offspring growth, from hatching success to early development traits (such as length, weight, SBIE). Additionally, differences in foraging capacity observed when males were crossed with D females suggest that these variations are more closely tied to domestication conditions, with males potentially playing a key role in influencing this phenomenon.

### Transcriptomic data

**Fig. 7:**
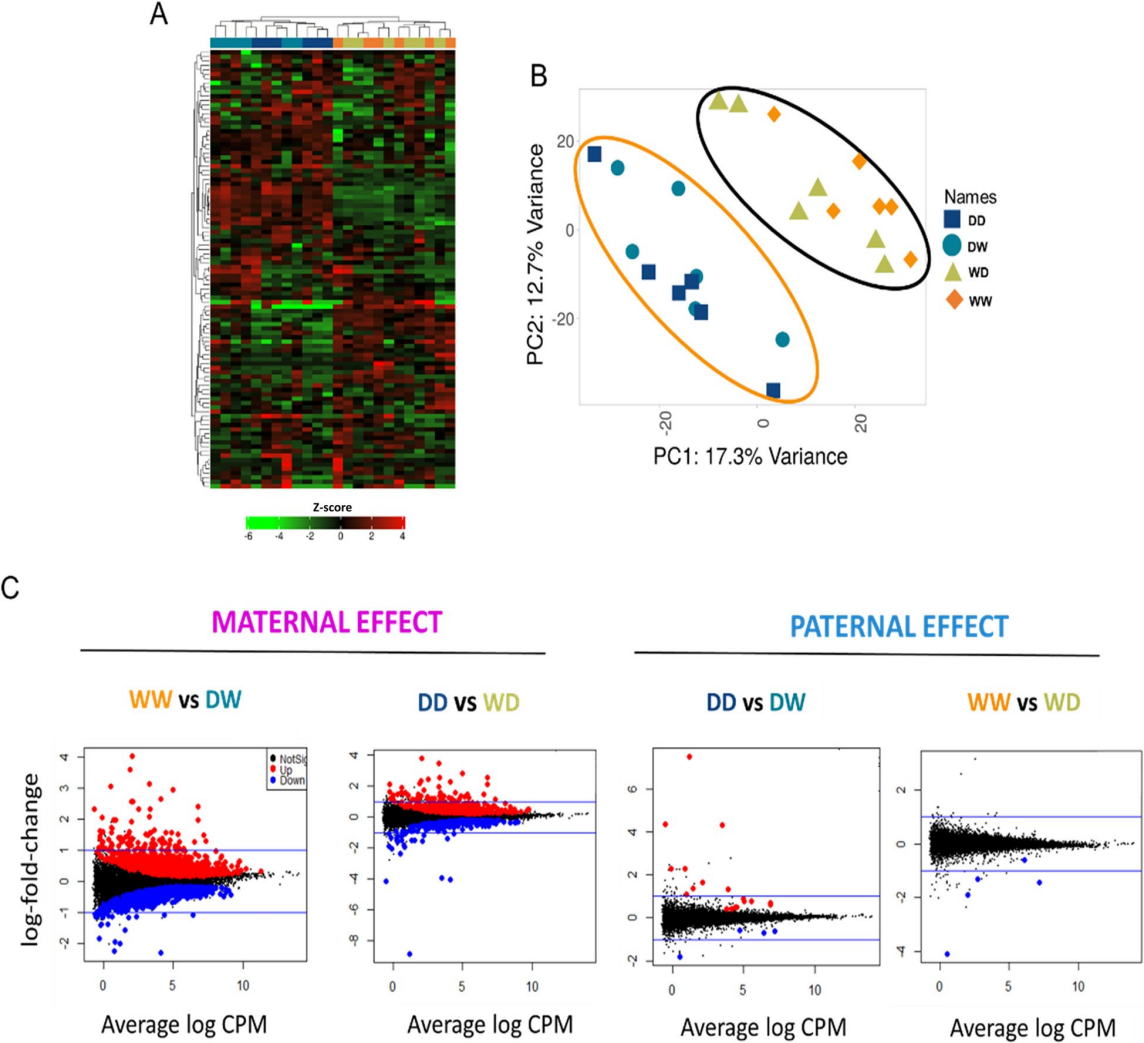
**A** Heatmap of RNA-Seq expression z-scores showing hierarchical non-supervised clustering of 100 most variable genes across experimental groups, presented as a descriptive visualization of transcriptomic patterns in freshly hatched Eurasian perch larvae. **B** Principal component analysis for all the Eurasian perch families created. The analyses were done on the basis of female origin — the orange ellipse depicts families created using domesticated mother, while the black ellipse depicts females from the wild origin. **C** Volcano plot of DEGs (FDR < 0.05) for all the 4 comparisons made for maternal- and paternal-effect analysis. CPM: counts per million

A differential expression gene (DEG) analysis was conducted to investigate maternal- and paternal effects across various comparisons. Following RNA-seq analysis, a total of 30,744 genes were initially identified. After applying filtering criteria for expression level, 20,447 protein-coding genes remained, which were then used for the DEGs analysis to identify potential specific maternal- and paternal-effect genes (for full list of DEGs see Additional file 2). Visualization of 100 most variable genes (Fig. 7A) shows a descriptive visualization of expression patterns and clustering among experimental groups and the principal component analysis (PCA) (Fig. 7B) reveals that the family distribution is clearly influenced by the female origin.

Around 17,000 identified and expressed genes were found to be non-differentially expressed, indicating that these genes consistently contribute to shaping the larvae’s profile, regardless of parental origin and can be named as “conserved genes”. A Gene Ontology (GO) analysis revealed that many of these non-differentially expressed genes are primarily associated with nervous system development and intracellular transport (see Additional file 1: Fig. S8).

For maternal effect analysis (with females as the variable and males as the constant) 2259 DEGs (FDR < 0.05) were found when females were crossed with W males, and 765 DEGs (FDR < 0.05) when paired with D males (Fig. 7C).

The analysis for paternal effect, with males being the variable and females as the constant, revealed 22 DEGs (FDR < 0.05) when males were crossed with D females, and only 5 DEGs (FDR < 0.05) when paired with W females (Fig. 7C).

**Fig. 8:**
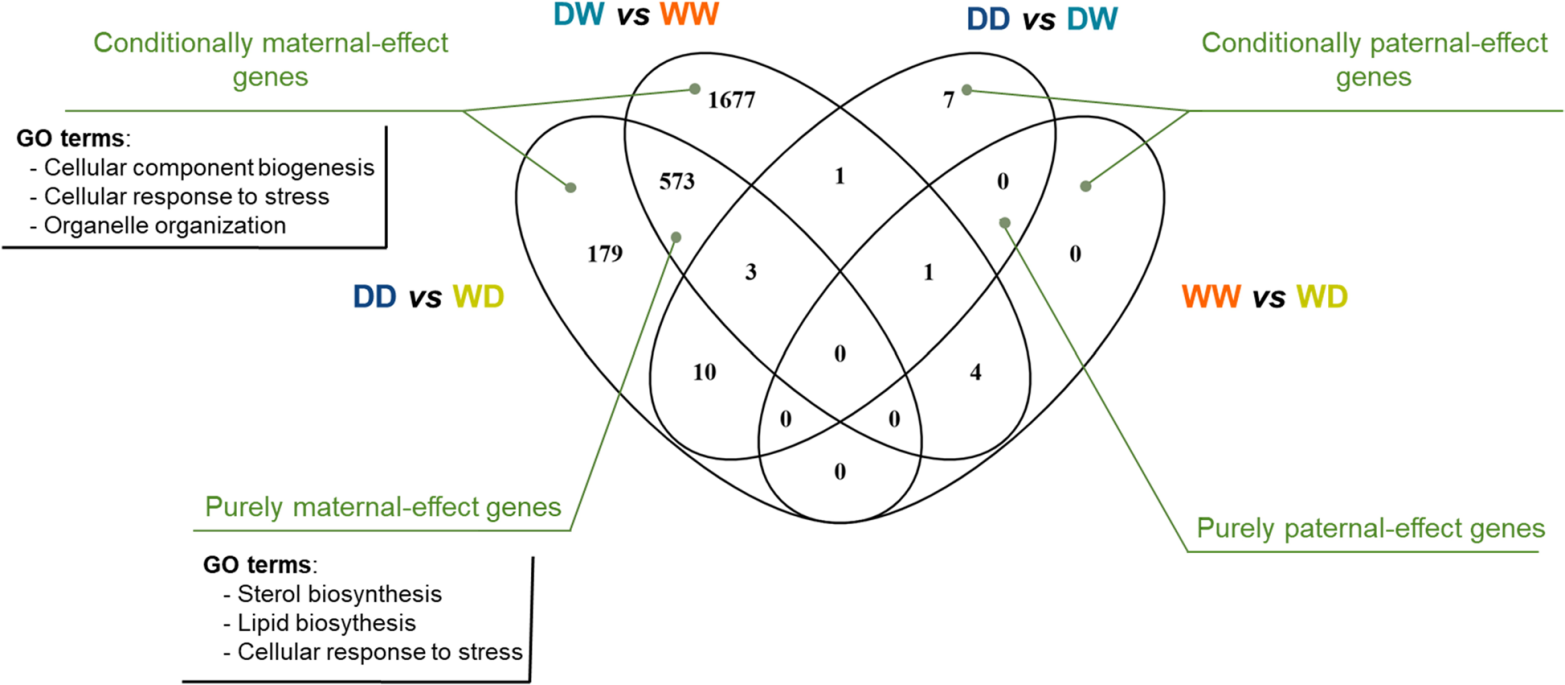
Venn diagram illustrating the overlapping genes classified as purely maternal-effect and paternal-effect genes, as well as those exclusive to conditionally maternal- and paternal-effect genes across different comparisons. Additionally, the figure lists the top three most enriched Gene Ontology (GO) terms associated with purely maternal-effect genes (*n* = 573) and all conditionally maternal-effect genes (*n* = 1856). D, domesticated; W, wild

To check for any overlap between groups, a Venn intersection analysis was performed (Fig. 8). This approach led to the identification of two categories of DEGs:

- purely maternal- or paternal-effect genes: which are common across groups regardless of the male’s (or females’) origin
- conditionally maternal- or paternal-effect genes: these genes show expression differences based on the specific paternal (or maternal) lineage. Their expression is influenced by interaction with a parent of a particular origin, indicating a conditional effect dependent on the paternal (or maternal) phenotype.

This analysis revealed that 573 genes were commonly shared across the maternal effect groups, regardless of the males’ origin; these genes were categorized as purely maternal-effect genes. Additionally, 179 genes were specific to groups where females were crossed with D males, while 1677 genes were specific to groups where females were crossed with W males. These genes were classified as conditionally maternal-effect genes. GO analysis revealed that purely maternal-effect genes are primarily involved in stress response processes, sterol and lipid biosynthetic processes (see Additional file 1: Fig. S7). In contrast, conditionally maternal-effect genes are mainly associated with regulation of cellular component biogenesis, cellular response to stress, DNA replication, and regulation of molecular functions (in Additional file 1: Fig. S6).

For the paternal-effect groups, no purely paternal-effect genes were identified. However, 7 genes were found when males were crossed with D females and these were classified as conditionally paternal-effect genes, listed here: *supt5h* (homolog, dsif elongation factor subunit); *znf648* zinc finger protein 648; *lamtor4* (late endosomal/lysosomal adaptor, mapk and mtor activator 4); *nlrc3* (nlr family card domain containing 3); *tim3* (T-cell immunoglobulin and mucin domain-containing protein 3); *pnmt* (phenylethanolamine n-methyltransferase); *LOC120549683* (interferon alpha-inducible protein 27-like protein 2A).

### Developmental expression profiles of perch-specific maternal-effect genes

**Fig. 9:**
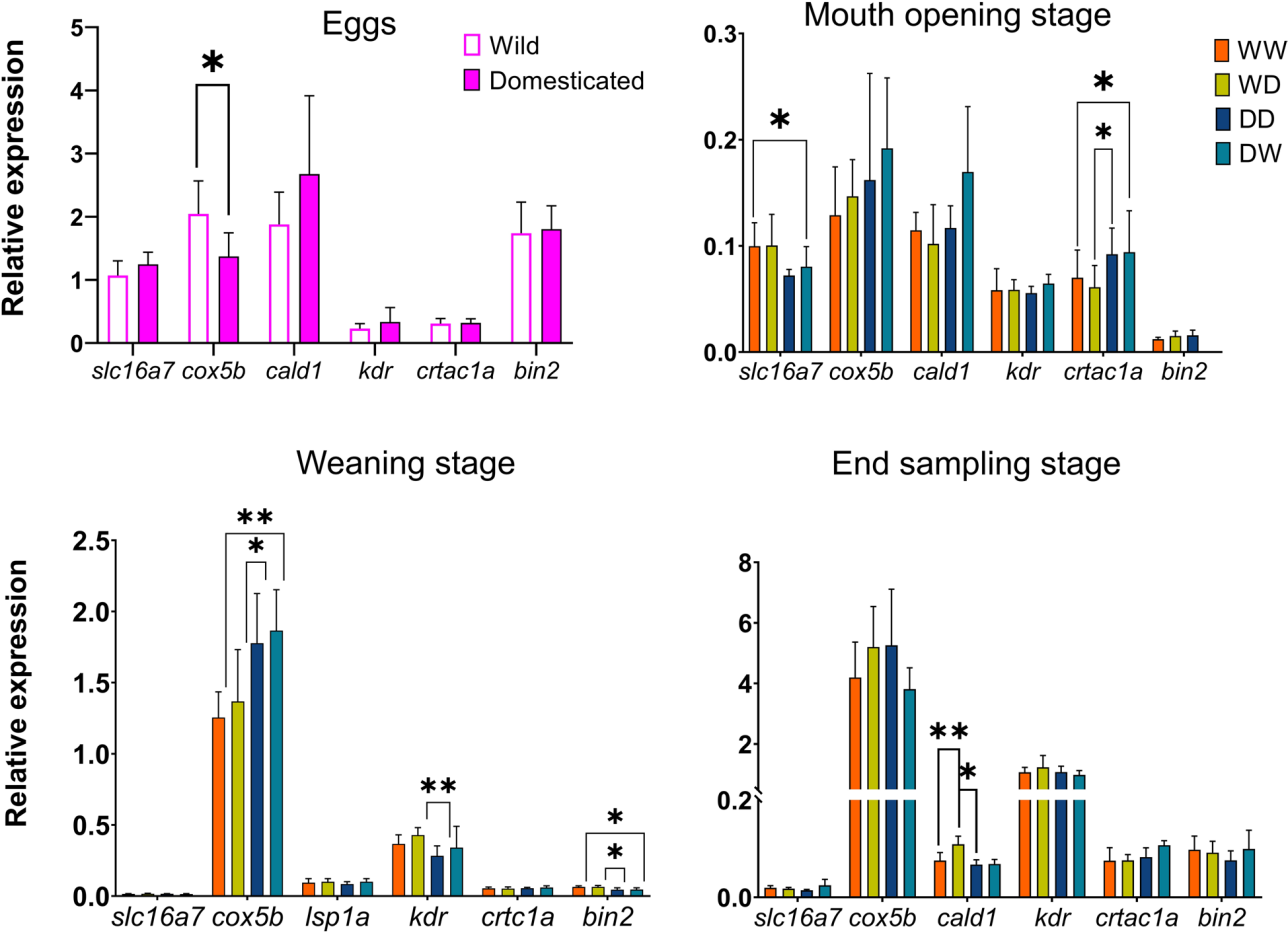
RT-qPCR for the 6 genes selected across the egg stage and various larval stages (from mouth opening to the end of the larval period, here referred to as end sampling). The results of statistical analysis are presented as follows: *p < 0.05, **p < 0.01. D, domesticated; W, wild. Error bars indicate standard deviation (SD)

We focused on a subset of the 573 purely maternal-effect genes to explore their maternal origin and potential perch-specific regulatory roles. Six candidate genes were selected based on their presence in the unfertilized egg transcriptome, their perch-specificity compared to zebrafish early development, and their high expression in perch eggs (Table 1, Fig. 9).

**Table 1:** Genes identified as Eurasian perch-specific maternal genes with maternal- effect documented in our study

Gene_id	Human orthologs	*Danio rerio* orthologs	Transcript_id	Full genes’ name
*crtac1a*	*CRTAC1A*	*crtac1a*	XM_039784574.1	Cartilage acidic protein 1
*LOC120558149*	*SLC16A7*	*slc16a7*	XM_039799069.1	Monocarboxylate transporter 2
*si:ch211-79k12.1*	*KDR*	*kdr*	XM_039806878.1	Vascular endothelial growth factor receptor 2
*LOC120547834*	*COX5B*	*cox2b*	XM_039783533.1	Cytochrome c oxidase subunit 5B, mitochondrial
*lsp1a*	*CALD1*	*cald1b*	XM_039809086.1	Caldesmon
*bin2b*	*BIN2*	*bin2a*	XM_039797340.1	Bridging integrator 2

All six genes were detected in unfertilized eggs, supporting their maternal origin (Fig. 9). Expression profiles tracked across developmental stages revealed diverse dynamics. For instance, *slc16a7* was highly expressed in eggs and mouth-opening larvae, then declined, suggesting an early developmental role. Interestingly, *cox5b* expression was initially higher in W eggs compared to D ones. However, in D samples, expression progressively increased throughout larval development, peaking at weaning — suggesting a sustained maternal influence. Other genes, such as *crtac1a*, *kdr*, and *cald1*, displayed stage- or cross-specific modulation, including late-stage increases or lineage effects.

Three of the six genes (*slc16a7*, *cald1*, *crtac1a*) showed expression at the mouth-opening stage consistent between RNA-seq and qPCR. Although full concordance was not observed across all genes, the multi-stage qPCR analysis offered insight into the temporal dynamics of these maternally inherited transcripts.

## Discussion

Understanding the respective contributions of maternal and paternal origins in shaping early life traits in Eurasian perch offers valuable insights into the underlying dynamics of larval development and performance. While parental origin influenced certain zootechnical traits, maternal effects consistently emerged as the primary driver of offspring phenotype, particularly during early developmental stages. This is likely due to the maternal provisioning of molecular and nutritional components within the egg, which directly support early growth, feeding capacity, and larval survival.

In contrast, the transcriptomic analysis of larvae at the mouth-opening stage revealed a more nuanced dynamic. While maternal influence remained predominant — reflected in the greater number of DEGs attributable to maternal origin — our results also point to subtle but detectable paternal contributions. Taken together, our findings suggest a regulatory framework in which maternally inherited transcripts establish the developmental foundation, while paternally inherited factors may modulate or refine gene expression, contributing to offspring condition in a context-dependent manner.

### Zootechnical traits

The results reveal that mothers have a dominant influence across several key zootechnical traits, such as hatching rates, growth-related traits (i.e., weight, length) and swim bladder inflation, while paternal effects has been limited to foraging kinetics only. This strong maternal impact is likely due to the female provisioning the egg with vital nutrients, hormones, and cytoplasmic components that sustain the larval development [36]. Importantly, the strong maternal influence observed in this study may reflect multiple underlying mechanisms. In addition to non-genetic inheritance through molecular provisioning, part of the observed variation may also be consistent with genetic maternal effects. Furthermore, environmentally induced maternal effects may also contribute, as maternal nutritional status, stress exposure, or environmental history prior to spawning can alter egg quality and composition, thereby shaping offspring performance. While our experimental design allowed us to identify a strong maternal effect, the underlying mechanisms cannot be fully resolved and may involve both genetic and non-genetic pathways.

Consistent with previous research [6, 37], we found that mothers play a critical role in shaping early larval performance, with length of larvae at mouth-opening (an important predictor of future performance and adaptability) emerging as a key example of a maternal-effect trait [28]. Notably, this strong maternal influence is particularly evident until oil droplet reduction stage. After this point, it appears to diminish as yolk’s oil droplets — containing the nutrients provided by the mother — are consumed, signalling a transition when larvae begin independent feeding [28]. This may explain why initially smaller or lower-weight wild larvae can compensate their growth once they shift to exogenous feeding. Overall, our results underscore the importance of maternal provisioning in early life stages, which appears to set the stage for growth and development, even if its direct influence seems to fade as larvae become self-sufficient. Further exploration could investigate whether these early maternal effects have any lasting impact on later stages of development or long-term performance outcomes.

Importantly, phenotypic differences between domesticated and wild populations are well-documented, with parental origin having a significant influence on offspring traits, particularly during the early post-hatching period [33–35]. This factor should therefore be carefully considered when interpreting the present results. Indeed, for certain traits, the domestication process appears to have enhanced performance. For example, larval groups originated from at least one domesticated parent exhibited superior foraging ability during first days of exogenous feeding compared to those from wild parents, with the latter experiencing higher mortality rates. Nevertheless, zootechnical results proved that the use of these two distinct populations (domesticated and wild), exhibiting strong phenotypic differences, served as a robust approach helping to better understand parental influence in shaping the offspring’s traits. These findings emerged despite the logistical and temporal differences in the spawning of wild and domesticated broodstocks. Rather than being confounding factors, these differences reflect biologically appropriate, highly optimized protocols that align with the long-term reproductive history of each stock. Importantly, these procedures were developed and refined over many years by the team members of this study. The resulting variation provided an opportunity to study parental effects under contrasting life-history conditions. We deliberately embraced this divergence to capture “extreme phenotypes”, thereby increasing our ability to detect meaningful parental contributions within a rigorously structured cross-design.

### Transcriptomics

In animals, parental contribution to the transcriptome is unequal and so is the control over early embryogenesis [38]. Recently, the transcriptomic profile of fish larvae at mouth-opening has been highlighted as a window to effectively reflect parental influences on offspring phenotypes [28]. In our study, we also focused our transcriptomic analysis on this developmental point, as it represents the end of embryogenesis and minimizes interfering effects from post-hatch environmental exposure. This allowed us to assess whether maternal dominance extends to the molecular level by analyzing gene expression patterns with attention to independent maternal and paternal contributions.

Based on the results, a profile of a freshly hatched larva (which in Eurasian perch coincide with the mouth-opening stage) would consist of approximately 80% of a conserved core of around 17,000 genes, displaying stable expression. These genes represent essential components for larval development, indicating that their expression at a certain level is crucial regardless of external factors. Many of these genes are linked to nervous system development and intracellular transport, both essential for larval growth and survival [39, 40]. Genes linked to neurogenesis are consistently expressed in the transcriptomes of larvae across diverse fish species [39, 41, 42], emphasizing the essential role of this process in early life history. The development of the nervous system is crucial, as it governs motor functions, sensory processing, and behavioural responses, all of which are vital for the proper adaptation and survival of fish larvae in their early stages [39]. Also, neurogenesis has been recognized as a non-genetically inherited process for embryonic development, with Colson et al. [43] and Żarski et al. [44] indicating a significant maternal influence on this pathway. The importance of neurogenesis-related genes underscores the importance of maternal inheritance, extending beyond genetic contributions to include regulatory non-genetic factors such as maternal mRNAs, which seem to play a key role in early neural development and functioning. Therefore, these findings suggest that a significant portion of the larval transcriptome remains robustly conserved, highlighting the stability of essential biological pathways crucial for embryonic and larval development. Additionally, the results indicate that suggested in the previous studies maternally derived modifications to nervous system development are more specific for particular neurogenesis pathways and processes, warranting further exploration.

Despite the conservation of a large percentage of the larval transcriptome, approximately 20% exhibits variability influenced by parental effects. Maternal effects, in particular, show a significantly higher number of DEGs compared to paternal effects. Notably, 573 genes were identified as purely maternal-effect genes, enriched in functions related to stress response, cholesterol biosynthesis, and ribosome biogenesis, suggesting a vital maternal role in preparing offspring for environmental challenges, metabolic demands, and growth. Similarly, conditionally maternal-effect genes (*n* = 1856) are predominantly involved in regulation of cellular components, cellular response to stress, DNA replication, and regulation of molecular functions. Their expression, however, appears to be adjusted by the experience (shaping non-genetic factors, such as epigenetic state) of the mating partner. This indicates a dynamic regulatory mechanism, where maternal inputs lay the foundation, while paternal inputs seem to refine gene expression, adjusting offspring phenotypes to suit specific environmental conditions or physiological challenges. Moreover, this set of genes can be seen as maternal-effect genes only when the male does not claim to govern them to act. This indicates that paternal contribution to gene expression is generally limited during early development, but may become active under specific conditions — potentially when paternal-origin signals (e.g., epigenetic marks or small RNAs acquired during the male’s life) influence gene regulation. Such effects might reflect environmentally mediated paternal programming or a form of conditional paternal influence that becomes relevant only under particular developmental contexts. Such dynamic interaction points to a complex regulatory mechanism, where both maternal and paternal influences interact in shaping offspring traits, highlighting the importance of both parents in early larval development and survival. These findings suggest a dynamic interplay scenario, where maternal and paternal factors interact to shape offspring characteristics.

The analysis of paternal-effect genes revealed 7 *conditionally paternal effect genes*. While these genes do not cluster under a single GO term, they are involved in critical biological processes, including stress responses, cellular growth, immune system regulation, and gene expression control, further suggesting that paternal influences also play a role in shaping the offspring’s phenotype, albeit in a more context-dependent manner. This confirms the notion that both maternal and paternal factors are integral to shaping early life stages, contributing to a balanced and adaptive developmental process.

Although this study did not identify any purely paternal-effect genes, likely reflecting the dominant role of maternal factors in the earliest stages of larval development, recent research suggests that paternal influences can still play a role. Specifically, sperm methylation patterns may affect offspring phenotypes, even if these effects are not immediately apparent in transcriptomic data [21, 30, 45]. For example, sperm DNA methylation patterns in Atlantic salmon have been shown to reflect environmental conditions experienced by males in captivity, with altered methylation correlating with fitness-relevant traits in F₁ offspring [46]. Similarly, brook charr exhibit paternal-line methylation influences that persist into fry stages and affect offspring growth and phenotype, particularly in response to parental thermal environment [47]. Moreover, in zebrafish, parts of the paternal methylome are retained through zygotic reprogramming, suggesting that sperm-derived epigenetic marks can functionally contribute during early development [48]. These findings collectively indicate that paternal effects may not manifest at the specific larval stage we analyzed but could emerge either later or under environmental triggers. Based on these studies, our observation — that purely paternal-effect genes are not present at the mouth-opening stage — does not exclude meaningful paternal influence later on. We therefore recommend future work explore epigenetic and gene expression patterns at earlier embryonic stages and during prolonged larval development, to fully capture the timing and impact of paternal contributions.

### Purely maternal genes

Maternal RNAs deposited in the eggs are critical to early embryonic development, yet the precise role and transfer mechanisms of specific maternal mRNAs from egg to offspring remain under investigation [49]. Our further validation strategy focused on following expression patterns of purely maternal-effect genes to pinpoint candidate Eurasian perch-specific maternal genes with further effect on offspring. This approach helped to elucidate various scenarios of maternal contributions from egg to juveniles.

One of the candidate genes, *crtac1*, is known for its role in chondrocyte differentiation and cartilage formation. It is an evolutionarily conserved gene across species, including fish [50]. Meanwhile, *slc16a7* encodes a monocarboxylate transporter (Mct2), vital for lactate and pyruvate transport [51], underscoring its importance in early larval metabolism when energy demands are high. Our study is the first to shed light on these genes as strong candidates for maternal-effect genes. Since we did not detect differential expression of these genes in unfertilized eggs (UFE), it may be suggested that maternal control starts later in the development with possible involvement of other genes interacting with them. This, however, requires further investigation by, at first, studying the kinetics of their expression along the embryonic development.

In the eggs, *cox5b*, which encodes a subunit of cytochrome c oxidase critical for mitochondrial respiration [52], showed higher expression in wild individuals compared to domesticated ones. Interestingly, this pattern reversed from the mouth-opening stage onward, with *cox5b* expression progressively increasing in domesticated larvae and peaking at weaning. This post-hatch upregulation suggests that domesticated individuals progressively enhance mitochondrial activity to support higher energy demands associated with rapid growth and the transition to exogenous feeding. These stage-specific shifts suggest a prolonged maternal influence on metabolic regulation, positioning *cox5b* as a strong candidate maternal-effect gene. Additionally, this supports the notion that transcriptomic profiling can serve as a predictive tool, providing insights into both the past conditions (maternal environment) and future developmental outcomes of offspring [28].

At the weaning stage, the *kdr* gene, also known as *vegfr2* (vascular endothelial growth factor receptor 2), showed a significant difference in expression, particularly in groups where females were crossed with domesticated males. This gene plays a crucial role in the early developmental stages of fish larvae, contributing to the formation of vascular networks essential for nutrient delivery and overall growth [53]. Considering our results, it is possible to speculate that vascular development may be particularly important during the weaning stage, as larvae transition from feeding on Artemia to dry feed, a dietary shift that imposes increased metabolic demands and necessitates efficient nutrient distribution via an enhanced vascular system. The observed higher expression of *kdr* in offspring from wild females compared to domesticated ones might relate to the offspring of wild females exhibiting accelerated growth starting at the weaning stage.

The expression of the *cald1* gene showed significant statistical differences only at the latest — juvenile — stage. This gene encodes caldesmon, a protein that plays a significant role in the regulation of actin and myosin interactions [54]. During the larval period, teleosts exhibit rapid growth and extensive changes in muscle structure [55]; therefore, this gene may play a crucial role in regulating actin-myosin interactions, essential for muscle contraction. Interestingly, in wild phenotypes, *cald1* expression in juveniles appears to be primarily under maternal control, whereas in the domesticated phenotype, it shifts during the larval period to paternal influence, highlighting another dimension of dynamic interplay between maternal and paternal effects during larval metamorphosis.

The *bin2* gene is a member of the BIN/amphiphysin/Rvs (BAR) family of proteins, which are involved in membrane dynamics, endocytosis, cytoskeletal interactions, neural network formation and immune cell regulation [56, 57]. Although its specific functions in fish larvae are not well characterized, the maternal influence observed at the weaning stage may relate to the critical physiological and immune system adjustments occurring during this period. Maternal factors, provided during oogenesis, seem to equip the larvae to better navigate developmental and environmental challenges encountered during the weaning transition. This highlights the importance of maternal contributions in preparing the progeny to overcome future developmental challenges.

This study aimed to explore how a subset of perch-specific maternal-effect genes behave throughout early ontogeny. While qPCR and RNA-seq were consistent for three of the six genes at the mouth-opening stage, the goal of the qPCR analysis was not to validate RNA-seq per se, but to gain insight into developmental expression patterns. Discrepancies between these platforms are well known and often reflect differences in sensitivity, transcript isoform detection, and reference normalization [58, 59]. Rather than a technical replication, the qPCR results allowed us to identify varying trajectories — from transient early expression (e.g., *slc16a7*), to persistent upregulation during the larval stages (*cox5b*), to reactivation at later stages (*kdr*). These findings add a dynamic, temporal layer to our understanding of how maternally derived transcripts may shape offspring development in perch.

In conclusion, the identification of candidate maternal-effect genes highlights the diverse physiological pathways influenced by maternal inheritance, ranging from metabolism and vascular development to muscle growth and immune regulation. Importantly, these genes should be interpreted as context-dependent candidates, rather than universal markers of maternal control, neither for Eurasian perch nor for other species. Nevertheless, these findings provide a foundation for exploring their broader implications in aquaculture and evolutionary biology. In a practical context, understanding maternal contributions to traits such as early growth and survival can inform broodstock selection and spawning strategies in perch culture, potentially improving larval performance and hatchery success.

## Conclusions

Basic rules of developmental process may seem straightforward — each parent provides genetic material to the progeny, equally contributing to its phenotype. However, as our results illustrate, this mechanism is far from simple. Equal genetic contribution does not always correspond to equal influence over the development of offspring. The analysis of the zootechnical performance highlights contributions showing dominant maternal control, shaping key aspects of progeny growth and survival. A deeper look into our results suggests that the shaping of progeny phenotype involves a dynamic and stage-dependent interplay between maternal and paternal contributions. While maternal inputs dominate early development, likely due to preloaded transcripts and egg provisioning, paternal influences appear later and may play a modulatory role in gene expression and phenotype refinement. This raises intriguing questions for selective breeding strategies: whether the selection of mothers over fathers should be prioritized, given their greater influence on offspring development. However, our findings reveal that parental effects are dynamic and growth-wise regulated, with maternal contributions playing a pivotal role during early stages, while paternal influences, though initially subtle and somewhat dormant, become increasingly significant during larval metamorphosis and beyond. This highlights a complex and dynamic interplay between maternal and paternal traits in shaping developmental trajectories. Rather than pointing to strict parental coordination [60, 61], our results suggest a temporally structured division of influence, where maternal factors dominate early development, while paternal contributions may gradually emerge later in ontogeny.

Despite the insights provided by this study, several limitations should be acknowledged. In particular, the transport and handling of broodstock and eggs before the experimental setup may have introduced additional stress-related or environmental influences that could have affected parental condition or early developmental processes. Although all efforts were made to standardize handling procedures (with temperature and oxygen levels strictly regulated to ensure comparability between groups), these factors cannot be completely excluded. Although the study focuses on non-genetic parental effects, it is important to recognize that not all observed gene expression differences necessarily reflect non-genetic inheritance, as some may arise from underlying genetic variation. While we aimed to disentangle maternal and paternal contributions, the complex and potentially interactive nature of parental effects makes it challenging to fully isolate the relative influence of each parent. Finally, the current study focuses on a single species and specific developmental window, which may restrict the generalizability of the findings across taxa and life stages. Therefore, future studies incorporating transcriptomic profiling of multiple developmental timepoints would be essential to capture these later-emerging paternal contributions and better understand the timing and transition of parental influence. Especially, that our results clearly suggest that this dynamic parental interplay over the progeny’s phenotype appears to begin at fertilization and persists throughout the embryonic and larval stages, continuing far longer than anticipated. Nevertheless, this work provides valuable insights into parental contributions to offspring phenotype and gene expression and offers a strong foundation for future studies.

## Methods

### Experimental design

Eurasian perch has been chosen as a model for this study due to its growing commercial importance as a valuable freshwater, non-salmonid teleost species. It serves as an ideal subject for research on fish domestication [62], reproduction [21, 63], development [64, 65], and physiology [11, 34], with easy access to wild and domesticated stocks [66]. Additionally, the establishment of standardized protocols for reproduction [67] and larviculture [34], alongside recent advancements in genomic research [21, 68] in this species, have enabled comprehensive investigations.

For this study, we created reciprocal crosses between wild (W) and domesticated (D) Eurasian perch, which represent biologically divergent parental types with well-documented phenotypic differences known to influence offspring characteristics [34, 35]. While these broodstocks differ in life history, our comparisons focused specifically on separating maternal and paternal effects, rather than testing for domestication effects. Their divergence served as a practical model to investigate parental contributions to offspring phenotype and gene expression. In this way, we intended to explore which traits and genes are under either maternal or paternal control in Eurasian perch larvae, regardless the mechanism of inheritance.

To accommodate natural and artificial reproductive cycles, the experiment was conducted in two phases: domesticated females were crossed in February under artificial photothermal conditions, while wild females were crossed in May during their natural spawning season. Cryopreserved milt from wild and domesticated males allowed consistent pairwise fertilizations across both time points.

**Fig. 10:**
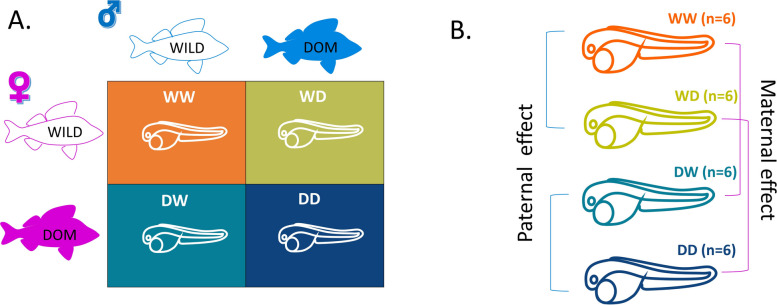
Conceptual experimental design and comparison framework. **A** Crossing matrix of wild (W) and domesticated (D) fish, resulting in four offspring experimental groups: wild females and wild males (WW), wild females and domesticated males (WD), domesticated females and wild males (DW), and domesticated females and domesticated males (DD). A colour scheme for each group has been kept consistent in the data visualization throughout the article. **B** Overview of the analysis strategy used for comparing groups to investigate maternal and paternal effects. DOM, domesticated

Each female’s egg ribbon was divided in two and fertilized with milt from a domesticated or wild male, generating four experimental groups: DD (domesticated♀ × domesticated ♂), DW (domesticated ♀ × wild♂), WW (wild♀ × wild♂), and WD (wild♀ × domesticated ♂) (Fig. 10A). This produced 24 families (6 per group). Pairwise analyses allowed us to isolate maternal effects (e.g., DD vs. WD; WW vs. DW) and paternal effects (e.g., DD vs. DW; WW vs. WD), with shared male or female partners across comparisons (Fig. 10B).

### Broodstock management and collection of gametes from domesticated and wild spawners

The broodstock characteristics for both wild and domesticated individuals are described in Additional file 3.

The domesticated fish used in the study belonged to the 8th generation bred under fully controlled (in indoor recirculating aquaculture system -RAS-) conditions at the Percitech fish farm in Switzerland. While, wild spawners were captured from lake Mikołajki (Poland) during the spawning season. The captured wild fish were transported in plastic bags with oxygen to the Center of Aquaculture and Ecological Engineering of the University of Warmia and Mazury in Olsztyn (CAEE-UWM, NE Poland) where they were placed in the RAS. In both cases, fish underwent consistent and standardized hormonally induced reproductive procedures described by Żarski et al. [69]. Briefly, both populations, during the spawning procedure, were kept at a controlled photoperiod with 14 h of light and 10 h of dark (14 L:10 D) and temperature (12 °C) until gametes collection. Fish were hormonally stimulated with a salmon gonadoliberin analogue (sGnRHa, BACHEM, Switzerland) with domesticated females being treated with two doses (10 and 25 µg kg^−1^ injection with 7-day interval) to promote and synchronize spawning in both sexes [69]. Wild females were treated with a single hormonal injection (50 µg kg^−1^). In both populations, milt was collected 7 days post hormonal stimulation (25 µg kg^−1^), which was within the optimal period of milt collection of this species [70]. Finally, eggs of domesticated fish were collected on day 9 following the priming injection, whereas eggs of wild females on day 4 after injection [71]. Prior to any manipulation, fish were anesthetized in MS-222 (Argent, USA) at a dose of 150 mg L^−1^.

The timing of spawning for domesticated (February) and wild (May) fish reflects the established reproductive biology of these two broodstocks. Domesticated perch were maintained under long-established artificial photo-thermal regimes used in commercial aquaculture, which induce predictable spawning windows aligned with hatchery operations. At Percitech, the breeding facility involved in this study, six genetically distinct broodstocks are maintained, each optimized to reproduce at different times of the year via tailored light and temperature cycles. In contrast, wild fish were spawned during their natural reproductive season in May. Importantly, the reproductive protocols used for both wild and domesticated fish have been independently optimized over many years by members of our research team, ensuring best-practice gamete handling and fertilization success tailored to each broodstock’s biology. While these protocols differed in timing and hormonal induction, they are not interchangeable. Applying a unified protocol across both groups would likely compromise gamete quality, fertilization rates, and embryonic viability.

### Milt collection and cryopreservation

For this study, from both populations cryopreserved milt was used to ensure procedural consistency. As mentioned earlier, this technique also enabled the novel approach of conducting pair-wise comparisons between the different families created.

Milt was stripped from 12 males, 6 domesticated (average weight 490.2 ± 91.5 g) and 6 wild (average weight 221 ± 86.6 g), by gently applying pressure to the abdomen, using a catheter (Galmed, Poland) to prevent contamination with urine or blood. After collection, each milt sample was kept on ice. Spermatozoa motility was first assessed using a two-step activation procedure. For fresh milt, samples were first diluted 1:50, and for frozen/thawed milt, they were diluted 1:5 in an immobilizing solution (150 mM NaCl, 5 mM KCl, 1 mM MgSO_4_ × 7H_2_O, 1 mM CaCl_2_ × 2H_2_O, 20 mM Tris, pH 8.0). Then, milt was then diluted 1:20 in an activating solution (75 mM NaCl, 2 mM KCl, 1 mM MgSO_4_ × 7H_2_O, 1 mM CaCl_2_ × 2H_2_O, 20 mM Tris, pH 8.0) with 0.5% bovine serum albumin.

Cryopreserved milt was essential to ensure controlled, repeatable fertilizations with the same male pairs across cross types, minimizing variation in gamete quality. This also allowed us to select families with the highest fertilization success, consistent with our prior studies emphasizing quality-controlled comparisons [44]. While domesticated males were larger, this reflected natural population differences and collection constraints. It is often difficult to collect wild males in sufficient number and size during their limited spawning season, and we used the best available individuals while aiming to minimize disparities. Rather than a confounder, size variation contributed to the desired contrast in parental traits, supporting our “extreme phenotype” approach.

Various motility parameters were measured using the Computer-Assisted Sperm Analysis (CASA) system, including motility (MOT, %), linearity (LIN, %), amplitude of lateral head displacement (ALH, µm), average path velocity (VAP, µm s^–1^), curvilinear velocity (VCL, µm s^–1^), and straight-line velocity (VSL, µm s^–1^) for both fresh and cryopreserved milt. CASA was conducted on fresh milt to establish baseline sperm quality at collection and to ensure standardized, high-quality samples entering the cryopreservation workflow. CASA was repeated post-thaw to quantify the functional impact of cryopreservation and to verify that thawed milt used for fertilization retained adequate motility. Additionally, the concentration of fresh milt was determined using the NucleoCounter SP-100 (Chemometec, Allerød, Denmark) [72]. Ensuring high-quality milt and accurately determining its concentration was crucial, as the cryopreservation procedure depends on the final sperm concentration, which is subsequently used to maintain a consistent sperm:egg ratio during in vitro fertilization.

Milt cryopreservation was carried out using the method developed by our Team [73] with a final concentration of 0.3 M glucose, 7.5% methanol and 25 mM KCl at 3 × 10^9^/ml spermatozoa.

### Egg collection and in vitro fertilization

Females were chosen on the basis of their oocyte maturation stages, in order to maintain synchronicity during rearing trials. Prior to hormonal treatment, females were evaluated by catheter-based oocyte sampling to determine oocyte maturation stage following established criteria. Briefly, oocyte maturation stage was evaluated according to the technique described by Żarski et al. [74] by catheterizing a sample of oocytes, exposing them in clarifying Serra’s solution (ethanol, formalin, and glacial acetic acid mixed 6:3:1 by volume) and microscopic assessment of their maturation stages based on 6-stage classification [71]. Ovulation was subsequently monitored via periodic gentle abdominal checks according to standard protocols for Eurasian perch [67].

**Fig. 11:**
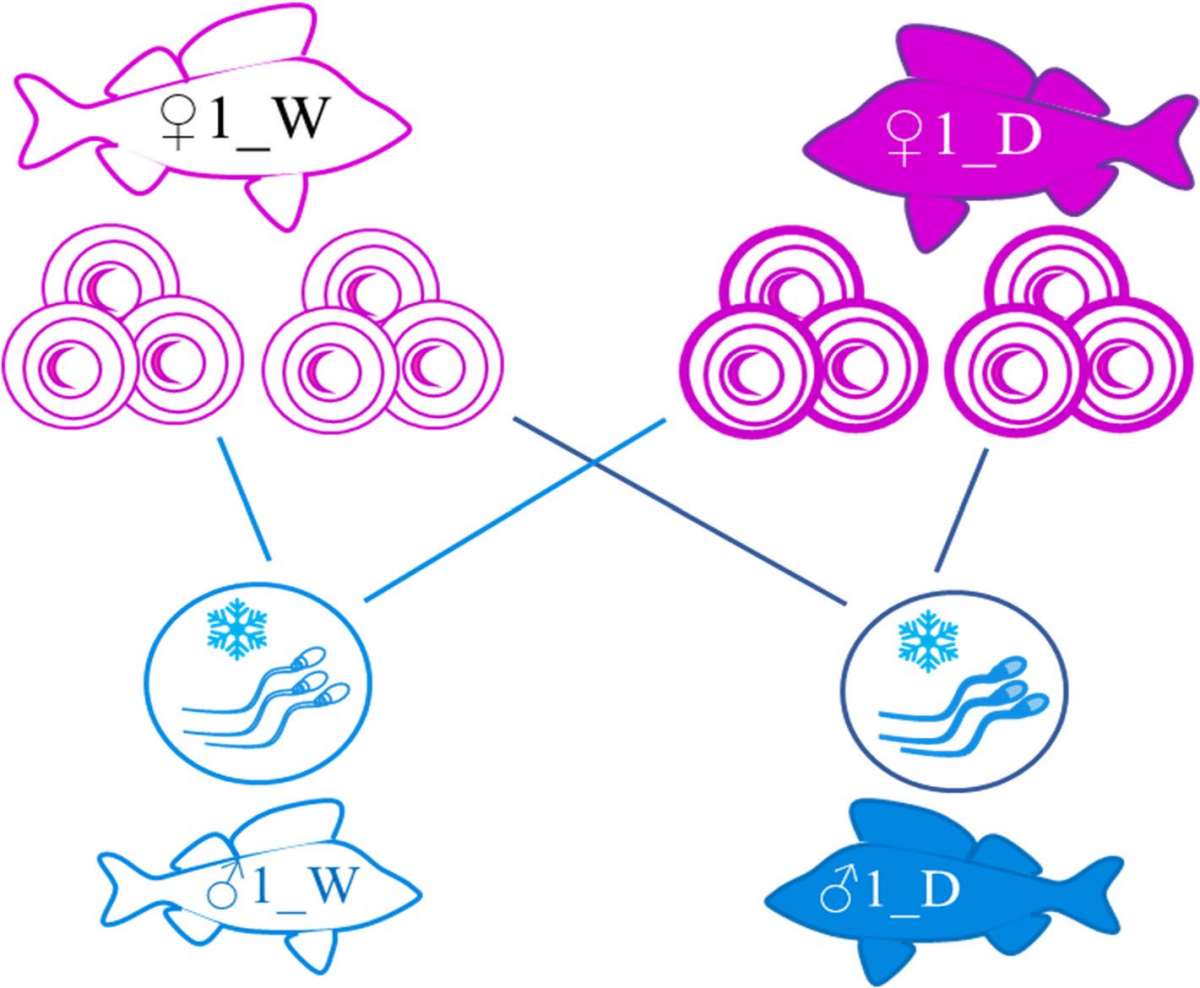
Paired fertilization design used to generate experimental crosses. The diagram illustrates a paired fertilization method, where eggs from a specific domesticated female (♀1_D) were fertilized in two ways: half with milt from a domesticated (♂1_D) male and the other half with wild male milt (♂1_W). The same combination of domesticated and wild males was used to fertilize eggs coming from a specific wild female (♀1_W). The same approach was followed for all the domesticated and wild females, using different combinations of males. W, wild; D, domesticated

Eggs were collected from 12 selected females: 6 domesticated (average weight 466 ± 81.4 g) and 6 wild (average weight 434.5 ± 102 g), using gentle abdominal pressure into a clean, dry beaker. Each egg ribbon, averaging 113 ± 25 g in weight (see Additional file 3), was then split into two equal parts. One part was fertilized with cryopreserved milt from a single domesticated male (e.g., ♂1_D), and the other with cryopreserved milt from a single wild male (e.g., ♂1_W). Importantly, each fertilization used milt from an individual male, not a pooled sample. The same male pairs were used to fertilize eggs from both domesticated and wild females (Fig. 11), ensuring consistency in paternal origin. This process was repeated for all 12 females, using different male combinations to create fully reciprocal crosses and paired samples for comparative analysis.

Just before in vitro fertilization, straws with milt were thawed in a water bath at 40 °C for 10 s and placed in an Eppendorf tube. Then, the eggs were preactivated for 30 s in Wyonarovich solution (1:5) [75], and milt was added to the eggs at a previously optimized sperm:egg ratio of 100,000:1 [72]. Upon introducing the thawed milt, eggs were then stirred for 30 s and washed with hatchery water after ~ 10 min to remove excess spermatozoa and any debris. Additionally, around 1 g of UFE were snap frozen in liquid nitrogen for further RNA extraction.

### Incubation of embryos

**Fig. 12:**
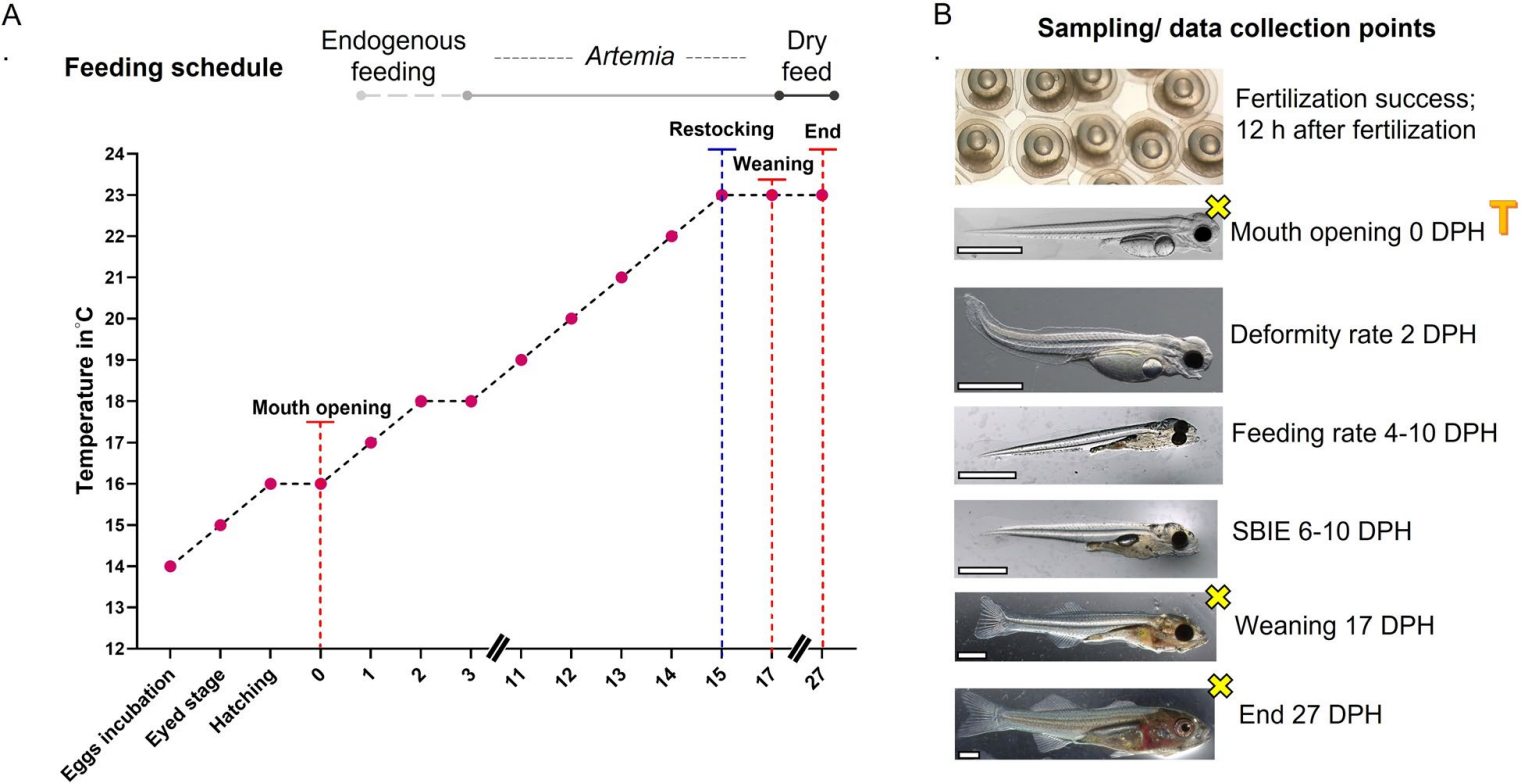
Protocol of eggs incubation and larvae rearing followed for the experiment. **A** Rearing schedule and temperature regimen used for embryos and larvae. **B** Sampling points for zootechnical data collection. Marked with yellow crosses are the sampling points for both zootechnical data collection and molecular analysis. T = describe the moment when collected larvae were used for transcriptomic analysis

The rearing trials for progeny derived from both wild and domesticated fish were carried out in the exact same way, with the exception that the fertilized domesticated eggs were transported in sealed plastic bags filled with water (70%) and oxygen (30%) and placed inside styrofoam boxes containing 500 g of ice to prevent overheating. The transport lasted approximately 16 h and covered a developmental window from mid-blastula to ~ 80% epiboly. Temperature was monitored at regular intervals and remained stable throughout, matching conditions used for wild embryos. Handling during transport was minimal, and the protocol followed procedures validated in previous studies involving perch (e.g., [34, 35]). While a minor influence of transport on early development cannot be entirely excluded, we consider it negligible given the short duration, stable conditions, and the biological consistency of transcriptomic and phenotypic outcomes. Next, upon arrival the fertilized eggs were further treated in exactly the same way as the eggs from wild fish. All the eggs were incubated in 15L tanks (which were later used for larval rearing) with black walls and upper water inflow, that functioned within the same RAS. The eggs were spread on mesh (diameter of around 3 mm) and kept in a water at a temperature of 14 °C (Fig. 12A). Fertilization rate (before embryos reached the mid-blastula transition) was calculated for each family separately (in duplicate), by counting ~ 100 embryos under the microscope. The photoperiod during all embryos’ incubation, and later larvae rearing (until 27 DPH, i.e., end of larval period) was maintained at 24L:0D (24 h light: 0 h dark; 1500 lx, measure at the water surface). When the embryos reached the eyed-egg stage, the temperature was raised to 15 °C; while, as soon as the first hatched larvae were noticed, the temperature in the system was raised to 16 °C. To maintain synchronous hatching, the larvae were hatched manually. This was done by transferring the egg ribbons to bowls with water from the rearing tanks and stirring gently. This operation was repeated few times until most of the larvae hatched. The day of hatching was considered as 0-day post-hatching (DPH).

After hatching, the larvae were left undisturbed for 24 h. On 2 DPH, they were counted volumetrically and distributed into three tanks (for each experimental group) at a density of 1500 larvae per tank.

### Larval rearing

At 1 DPH, the water temperature was raised to 17 °C, and at 2 DPH to 18 °C, which was kept stable up to 10 DPH. From 11 DPH onward, the water temperature was gradually increased by 1 °C per day until 23 °C, considered the optimal temperature for the growth of Eurasian perch larvae [28, 76]. Starting from 3 DPH, the mortality of the larvae was counted twice a day after each cleaning. From 4 DPH, larvae were started to be fed with Artemia sp. nauplii ad libitum three times per day (first 4 days of feeding — micro Artemia cysts [SF origin], then standard size Artemia cysts at 260,000 nauplii per gram [GSL origin]) [77] (Fig. 12A). Ad libitum feeding was operationally defined as maintaining a slight excess of viable *Artemia nauplii* between feeding events, verified by visual inspection of prey availability and larval gut fullness. Artemia quantities were standardized across tanks and experiments to ensure consistency between experimental groups. The deformity rate was counted at 2 DPH (Fig. 12B). Also, from 4 DPH, feeding rates started to be monitored by randomly collecting ~ 100 larvae from each tank and examining their stomachs under a stereoscopic microscope (Leica, Germany) to determine whether they contained food. Starting at 6 DPH, swim bladder inflation effectiveness (SBIE %) was measured in the same way as feeding rates. Both feeding rates and SBIE were recorded until 10 DPH, at which larvae had finally yolk sac fully utilized. After this time, no increase in the number of larvae with filled swim bladders was observed, while larvae that had not begun eating by this time already died. In addition, from 12 DPH, dead larvae were observed under the microscope to evaluate type I cannibalism (when the prey is partially ingested) (Kestemont et al. 2003).

Two days before weaning (15 DPH) larvae from each family were counted manually and restocked in the density of 500 larvae/tank. This was done to ensure the same number of larvae, which varied due to the different mortality rates observed in some families. After weaning (that took place at 17 DPH) larvae were fed exclusively with dry feed (Perla Larva Proactive, Skretting, Norway) three times a day, sprinkling it into each tank in small amounts for ~ 15 min each time. Oxygen level in the tanks was checked every day (with Aquaculture oximeter Polaris) and it was never below 80% concentration along with ammonia and nitrites concentration every 2 days (using DR1900 Portable Spectrophotometer), and it was never higher than < 0.02 mg L^–1^. The experiment ended on 27 DPH when more than 50% of the larvae showed no fin fold, therefore more than half of the fish finished already the larval period.

### Sampling points

Zootechnical traits of larvae were evaluated based on data obtained during three precisely chosen developmental moments following Palińska-Żarska et al. [34] and Debernardis et al. [28]:

1. At the mouth opening stage (0 DPH) — where at least 50% of larvae were found to have their mouth open. This is the point at which the larva is ready for independent life, while still being subjected to minimal manipulation by humans and the conditions in which it lives.
2. At the moment of weaning (17 DPH) — the moment when larvae start to be fed with compound diets,

3. At 27 DPH — considered the end of the larval period (when at least 50% of larvae finish their larval stage) and the end of the experiment.

At each of these sampling points, *n* = 30 larvae per family (*n* = 10 from each tank) were collected to measure total length (TL, ± 0.01 mm) and wet body weight (WBW, ± 0.1 mg). Additionally, the yolk sac volume (V, ± 0.01 mm^3^) of freshly hatched larvae was calculated using the spheroid formula, V = π/6 × l × h^2^, where *l* is the yolk-sac length and *h* is the yolk-sac depth. The larvae were first anesthetized using MS-222 at a concentration of 150 mg L^−1^, then photographed under a stereoscopic microscope (Leica, Germany) for TL measurement, while WBW was determined using a precision laboratory scale by placing the anesthetized larvae on a nylon net (mesh size approx. 200 μm) and gently blotting excess water with filter paper [78]. At each sampling point, additional *n* = 30 larvae from each family were collected and preserved in RNAlater (Sigma-Aldrich, Germany) for subsequent molecular analyses.

### RNA extraction

Total RNA was extracted from snap frozen UFE (~ 50 eggs) and larvae at three different developmental stages (mouth-opening stage, weaning and at the end of larval period) using a TotalRNA mini-kit (A&A Biotechnology, Poland). Specifically, for each family, RNA was extracted from a pool of 10 larvae at mouth opening stage (10.12 ± 0.17 mg). For larvae at the weaning stage, RNA was isolated from a pool of four larvae per family (77.62 ± 4.29 mg), and for larvae at the end of larval stage, from a pool of three larvae per family (208 ± 17 mg). After extraction the concentration and purity of the RNA were assessed with a DS-11 spectrophotometer (Denovix), showing absorbance ratios of A260/280 ≥ 2.0 and A260/230 ≥ 2.2. Quality of the RNA was further confirmed using the Agilent Bioanalyzer 2100 (Agilent Technologies, USA), with all samples exhibiting RIN values ≥ 9.0.

Importantly, only RNA samples from larvae at mouth opening stage were then sent for transcriptomic analysis. RNA extracted from UFE and other sampling points were used for real-time qPCR validation.

### RNA sequencing and library preparation

Twenty-four different libraries were created [79]. RNA-seq analysis was performed by Macrogen (Amsterdam, Netherlands) using the TruSeq Stranded mRNA kit (Illumina) with a NovaSeq6000 platform, and 60 M 150 bp paired-end reads per sample were generated. Read files have been processed with nf-core/rnaseq v3.12.0 [80] using GCF_010015445.1_GENO_Pfluv_1.0_genomic.fna as reference genome and GCF_010015445.1_GENO_Pfluv_1.0_genomic.gtf as reference annotation with “–skip_biotype_qc” and “–aligner star_rsem” parameters. Briefly, the reads were checked with fastqc [81] and trimmed with trimgalore [82] then aligned to the reference genome with STAR [83] and quantified with RSEM [84]. A summary table with general statistics of the RNA-Seq data is provided in Additional file 4.

### Differential expression gene (DEG) analysis

RNA-seq data were analysed by performing comparisons both between and within subjects using the edgeR package in RStudio, following the authors’ recommendations [85, 86]. Low-expressed genes were filtered by *filterByExpr* function, as advised. To analyse maternal effects, females were treated as the variable factor, while males, whether domesticated or wild, were kept constant (DD vs WD and WW vs DW). Likewise, for the assessment of paternal effects, males were considered the variable factor, with females held constant throughout the analysis (DD vs DW and WW vs WD). Differences were considered significant when false discovery rate (FDR) was inferior to *ɑ* (*ɑ* = 0.05) and specific differentially expressed genes (DEGs) were found for maternal and paternal effects comparisons.

Next, the DEGs found for the different experimental groups were compared to identify specific genes unique to each comparison as well as those shared between them, and the results were visualized using a Venn diagram [87].

To explore the variability in transcriptomic data, a heatmap showcasing the top 100 most variable genes and a principal component analysis (PCA) plot have been generated using iDEP 2.0 for visualization [88]. The 100 most variable genes were defined as those with the highest variance in normalized expression values across all samples.

### Gene Ontology (GO) analysis

GO analysis was conducted following the method described by Żarski et al. [44]. In brief, Eurasian perch transcriptome was first mapped to the human proteome using the Swiss-Prot database. Sequence alignment was then performed using BLASTX, and only the top match for each protein was selected, providing gene names and UniProt accession numbers for the aligned proteins. These were subsequently utilized for GO analysis using the ShinyGO platform [89]. GO analysis was carried out on DEGs found, separately for each comparison. The 20 most enriched biological processes were identified based on an FDR < 0.05.

### In silicoidentification of perch-specific maternal-effect genes

**Fig. 13:**
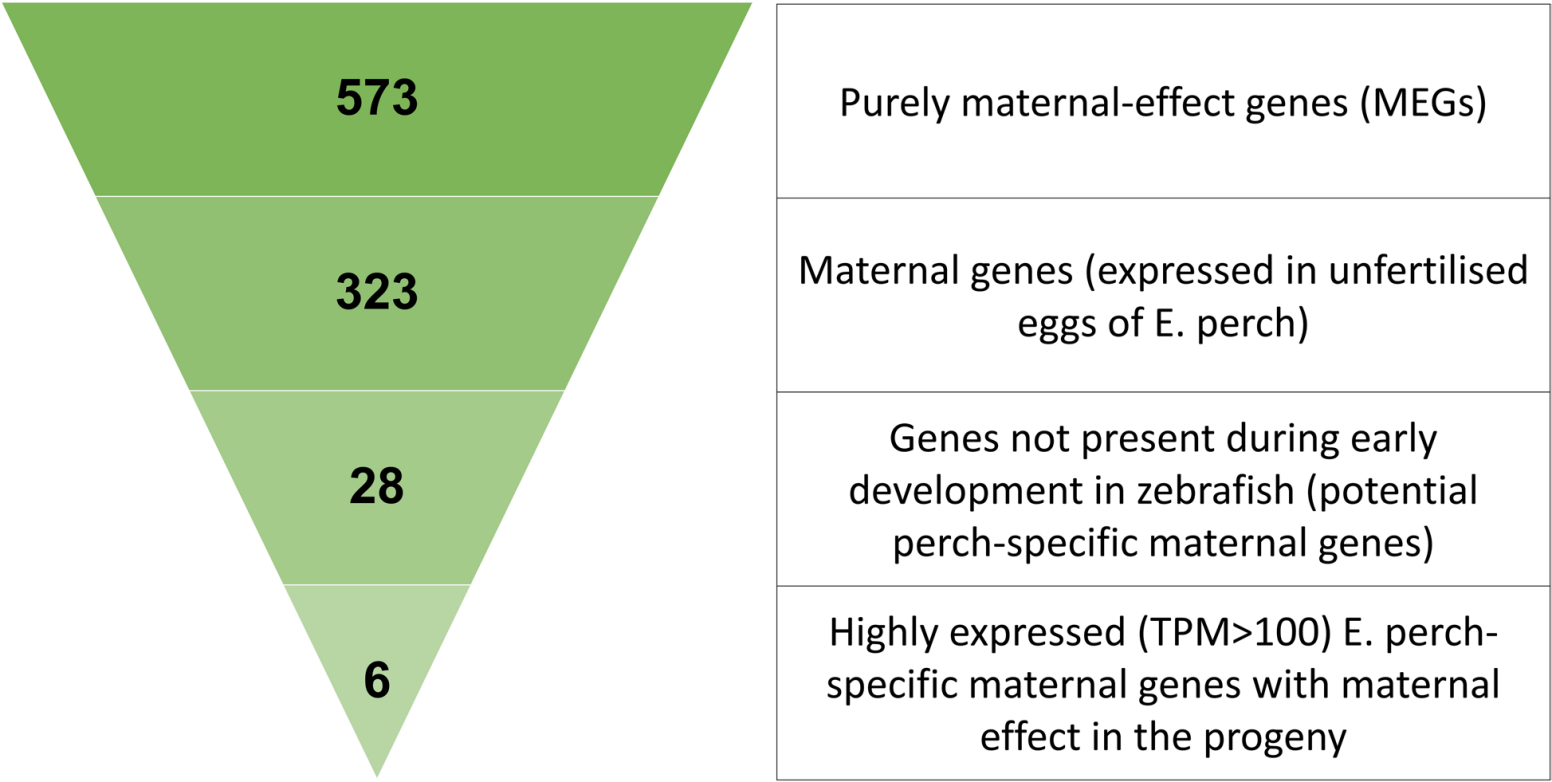
Graphic representation of the filtering cascade used to select the six Eurasian-perch-specific maternal genes with documented maternal-effect candidates

The direct transfer of maternal RNAs from egg to progeny remains a topic of ongoing debate. This study focuses on RNAs, with one of the aims being to investigate the presence of maternal RNAs in offspring. To explore this, we sought to identify gene candidates that could serve as maternal effect genes specific for Eurasian perch. In detail, out of the 573 identified purely maternal-effect genes (for details see the “Results” section), 324 DEGs were found to be present in the UFE transcriptome of Eurasian perch [21], what enabled to identify maternal genes (i.e., they were deposited as maternal transcripts in the egg) (Fig. 13). To explore how selected maternal-effect genes (MEGs) behave throughout early development, we carried out a biologically informed in silico filtering to select a subset of candidates for further expression trajectory analysis. Among the 573 purely maternal-effect genes identified by RNA-seq, we first retained 324 genes also present in the unfertilized egg (UFE) transcriptome [21], suggesting direct maternal RNA deposition. To identify perch-specific MEGs, we compared these transcripts to zebrafish expression profiles (Expression Atlas; [90]), focusing on expression between zygote and mid-blastula stages. Genes with no detectable expression in zebrafish (TPM < 0.5) were considered potentially perch-specific and maternally derived. This yielded 28 candidate genes, of which the six most abundant in perch eggs (> 100 TPM) were selected for developmental expression profiling across larval stages (Fig. 13, Additional File 2). These genes were not selected to validate RNA-seq outcomes statistically, but to assess whether such perch-specific maternal transcripts exhibit distinct persistence or regulation during early development.

### Exploration of expression profile of candidate maternal-effect genes during early development by RT-qPCR

Total RNA from eggs and larvae were reverse transcribed using the TranScriba kit (A&A Biotechnology, Poland) with oligo(dT)18 primers according to the manufacturer's instructions. Briefly, 1 µg of total RNA was mixed with 4 µl of 5 × reaction buffer, 0.5 µl of RNAse inhibitor, 2 µl of dNTP mix and 4 µl of TranScriba reverse transcriptase. The reaction was conducted for 60 min at 42 °C and then completed by heating at 70 °C for 5 min.

Primers for the 6 selected genes along with 6 reference genes for RT-qPCR were designed using Primer3Plus software version 3.3.0 [91]. For egg samples, two common reference genes were employed: beta actin (*b-actin*) and ribosomal protein L8 (rpl8). For larval samples, the reference genes included: ATP Synthase Peripheral Stalk-Membrane Subunit B (*atp5pb*)*,* Nascent Polypeptide Associated Complex Subunit Alpha (*naca*)*,* ATP Synthase F1 Subunit Gamma (*atp5f1c*)*,* Isocitrate Dehydrogenase (NAD ( +)) 3 Non-Catalytic Subunit Beta (*idh3b*). These reference genes were selected based on their lowest coefficient of variation (CV) recorded in our transcriptomic data [44], using TPMs calculated for all the biological replicates. The sequences of the designed primers are presented in Additional file 5.

Real-time qPCR was then conducted using a Viia7 thermocycler (Applied Biosystems). For each qPCR reaction (20 µL total volume), 10 ng of cDNA template was combined with SYBR Green qPCR Master Mix (A&A Biotechnology, Poland) and 0.5 µM of both forward and reverse primers. The cycling conditions consisted of a 10-min enzyme activation at 95 °C, followed by 40 cycles of denaturation at 95 °C for 15 s, and annealing and elongation at 60 °C for 1 min. After amplification, the efficiency of each primer was calculated using the Real-time PCR Miner program [92]. Then, the changes in gene expression were analysed using the delta delta Ct (2^–ΔΔCt^) method [93]. Data were normalized using geometric mean of reference genes.

### Data analysis and statistics of zootechnical traits

The analysis of zootechnical data was performed using R studio (version 4.3.2). Weight, length, and yolk sac volume were analysed with linear mixed-effects models (LMM) implemented in lme4 package, including female genotype, male genotype, and their interaction as fixed effects, and parental identity as a random effect. Fertilization, hatching, deformity rates, mortality (before and after restocking), and cannibalism rate were analysed using zero-inflated beta regression mixed-effects models fitted with the glmmTMB package. All models included female phenotype, male phenotype, and their interaction as fixed effects, with male or female identity included as a random effect. Cumulative and daily mortality rate, feeding rate and SBIE, which were measured repeatedly over multiple days, were analysed using repeated-measures mixed-model ANOVA; with day, female phenotype, male phenotype, and their interactions as fixed effects, and female and male identities as random effects; day was treated as a categorical factor. Estimated marginal means and Tukey-adjusted pairwise comparisons were computed using emmeans package. Differences were considered statistically significant when *p* < 0.05 (detailed results of the statistical analysis are provided in Additional file 6). Graphs were generated using GraphPad Prism (version 9.4.1). Importantly, the biological replicates (*n* = 6) within each cross were considered the independent units, and to ensure robust estimation of family means, a minimum of 30 individuals were measured per replicate.

## Supplementary Information

Additional file 1: Fig. S1–S8. Fig. S1. Deformity and cannibalism rate (%) and yolk sac (mm^3^) measured for all the Eurasian perch families. Asterisks show significance difference (*p* < 0.05). Fig. S2. Cumulative mortality (mean ± SD) before and after restocking for all crossings of Eurasian perch larvae analysed for maternal-effect. Error bars indicate standard deviation (SD). Asterisks show significance difference (*p* < 0.05, **p < 0.01, ***p < 0.001). Fig. S3. Cumulative mortality (mean ± SD) before and after restocking for all crossings of Eurasian perch larvae analysed for paternal-effect. Error bars indicate standard deviation (SD). Asterisks show significance difference (**p < 0.01, ***p < 0.001). Fig. S4: Daily mortality (mean ± SD) before and after restocking for all crossings of Eurasian perch larvae analysed for maternal-effect. Error bars indicate standard deviation (SD). Asterisks show significance difference (**p < 0.01, ***p < 0.001). Additional statistical information is provided in Additional File 6. Fig. S5: Daily mortality (mean ± SD) before and after restocking for all crossings of Eurasian perch larvae analysed for paternal-effect. Error bars indicate standard deviation (SD). Asterisks show significance difference (**p < 0.01, ***p < 0.001). Additional statistical information is provided in Additional File 6. Fig. S6. Tree view and network visualization showing the 20 most significantly enriched GO (biological process) for non-differentially expressed genes. Fig. S7. Tree view and network visualization showing the 20 most significantly enriched GO (biological process) for purely maternal-effect genes. Fig. S8. Tree view and network visualization showing the 20 most significantly enriched GO (biological process) for all the conditionally maternal-effect genes.

Additional file 2: Table S1–S5. Table S1. List of 22 DEGs (FDR < 0.05) resulted from the paternal-effect analysis where males were crossed with domesticated females. Table S2. List of 5 DEGs (FDR < 0.05) resulted from the paternal-effect analysis where males were crossed with wild females. Table S3. List of 765 DEGs (FDR < 0.05) resulted from the maternal-effect analysis where females were crossed with domesticated males. Table S4. List of 2259 DEGs (FDR < 0.05) resulted from the maternal-effect analysis where females were crossed with wild males. Table S5. A table presenting the expression patterns of 28 genes in zebrafish, spanning from the zygote stage to the early larval stage. The final column displays the average expression levels of these genes in the E. perch unfertilised egg transcriptome. Six genes, highlighted in the table, show notably high expression levels (> 100 TPM) in E. perch eggs, compared to zebrafish.

Additional file 3: Broodstock characteristics for both wild and domesticated individuals and milt evaluation with CASA after cryopreservation. WBW: Wet Body Weight, LT: Total Length; W: Weight. ALH: Amplitude of Lateral Head Displacement; LIN: Linearity; VAP: Average Path Velocity; VCL: Curvilinear Velocity; VSl: Straight Velocity; MOT: percentage of sperm motility.

Additional file 4: General RNA-seq Statistics. Duplnt: Intercept value from DupRadar; % Dups: Mark Duplicates—Percent Duplication; M Aligned: Reads Aligned (millions); % Alignable: % Alignable reads; % Proper Pairs: % Reads mapped in proper pairs; Error rate: Error rate: mismatches (NM) / bases mapped (CIGAR); M Non-Primary: Non-primary alignments (millions); % Mapped: % Mapped Reads; % Proper Pairs: % Properly Paired Reads; M Total seqs: Total sequences in the bam file (millions); M Reads Mapped: Reads Mapped in the bam file (millions).

Additional file 5: List of the primers used for RT-qPCR in the study. * = housekeeping genes.

Additional file 6: Full statistical results for zootechnical traits, including linear mixed-effects models and generalized linear mixed-effects models with post hoc comparisons.

## Data Availability

Raw RNAseq data of different families of freshly hatched larvae have been deposited in the NCBI Sequence Read Archive (SRA) under the BioProject accession number PRJNA1196822 (ID: 1196822) (https://www.ncbi.nlm.nih.gov/bioproject/PRJNA1196822/). All datasets are publicly accessible, and additional information is available from the corresponding author upon reasonable request.

## Abbreviations

ALH: Amplitude of lateral head displacement
*atp5f1c*: ATP Synthase F1 Subunit Gamma
*atp5pb*: ATP Synthase Peripheral Stalk-Membrane Subunit B
*b-actin*: Beta actin
*bin2a*: Bridging integrator 2
CaCl2: Calcium chloride.
*cald1b*: Caldesmon
CASA: Computer-Assisted Sperm Analysis
*cox2b*: Cytochrome c oxidase subunit 5B, mitochondrial
*crtac1a*: Cartilage acidic protein 1
CV: Coefficient of variation
D: Domesticated
DD: Domesticated ♀ × domesticated ♂
DEG: Differential expression gene
DPH: Days post hatching
DW: Domesticated ♀ × wild♂
FDR: False discovery rate
GLMMs: Generalized linear mixed-effects models
GO: Gene Ontology
H2O: Water
*idh3b*: Isocitrate Dehydrogenase (NAD ( +)) 3 Non-Catalytic Subunit Beta
KCl: Potassium chloride
*kdr*: Vascular endothelial growth factor receptor 2
L:D: Light and dark
LIN: Linearity
LMMs: Linear mixed-effects models
MEGs: Maternal-effect genes
MgSO4: Magnesium sulfate
MOT: Motility
*naca*: Nascent Polypeptide Associated Complex Subunit Alpha
NaCl: Sodium chloride
NGI: Non-genetic inheritance
PCA: Principal component analysis
RAS: Recirculating aquaculture system
*rpl8*: Ribosomal protein L8
RT-qPCR: Real Time Quantitative Polymerase Chain Reaction
SBIE: Swim bladder inflation effectiveness
sGnRHa: Salmon gonadoliberin analogue
*slc16a7*: Monocarboxylate transporter 2
TL: Total length
TPM: Transcripts per million
Tris: Tris(hydroxymethyl)aminomethane
UFE: Unfertilised eggs
VAP: Average path velocity
VCL: Curvilinear velocity
VSL: Straight-line velocity
W: Wild
WBW: Wet body weight
WD: Wild♀ × domesticated ♂
WW: Wild♀ × wild♂
