# Supplementary material for: Dynamic interplay of maternal and paternal contributions to offspring phenotype in Eurasian perch
Source: BMC Biol. 2026 Apr 20;24:129. doi: 10.1186/s12915-026-02602-x (PMC13224677; doi:10.1186/s12915-026-02602-x)
Supplement: Supplementary file 1 — Additional file 1: Fig. S1–S8. Fig. S1. Deformity and cannibalism rate (%) and yolk sac (mm3) measured for all the Eurasian perch families. Asterisks show significance difference (p < 0.05). Fig. S2. Cumulative mortality (mean ± SD) before and after restocking for all crossings of Eurasian perch larvae analysed for maternal-effect. Error bars indicate standard deviation (SD). Asterisks show significance difference (p < 0.05, **p < 0.01, ***p < 0.001). Fig. S3. Cumulative mortality (mean ± SD) before and after restocking for all crossings of Eurasian perch larvae analysed for paternal-effect. Error bars indicate standard deviation (SD). Asterisks show significance difference (**p < 0.01, ***p < 0.001). Fig. S4: Daily mortality (mean ± SD) before and after restocking for all crossings of Eurasian perch larvae analysed for maternal-effect. Error bars indicate standard deviation (SD). Asterisks show significance difference (**p < 0.01, ***p < 0.001). Additional statistical information is provided in Additional File 6. Fig. S5: Daily mortality (mean ± SD) before and after restocking for all crossings of Eurasian perch larvae analysed for paternal-effect. Error bars indicate standard deviation (SD). Asterisks show significance difference (**p < 0.01, ***p < 0.001). Additional statistical information is provided in Additional File 6. Fig. S6. Tree view and network visualization showing the 20 most significantly enriched GO (biological process) for non-differentially expressed genes. Fig. S7. Tree view and network visualization showing the 20 most significantly enriched GO (biological process) for purely maternal-effect genes. Fig. S8. Tree view and network visualization showing the 20 most significantly enriched GO (biological process) for all the conditionally maternal-effect genes. [file 12915_2026_2602_MOESM1_ESM.pdf]

Additional file 1: Supplementary figures related to zoothecnical traits results and the gene ontology (GO) analysis

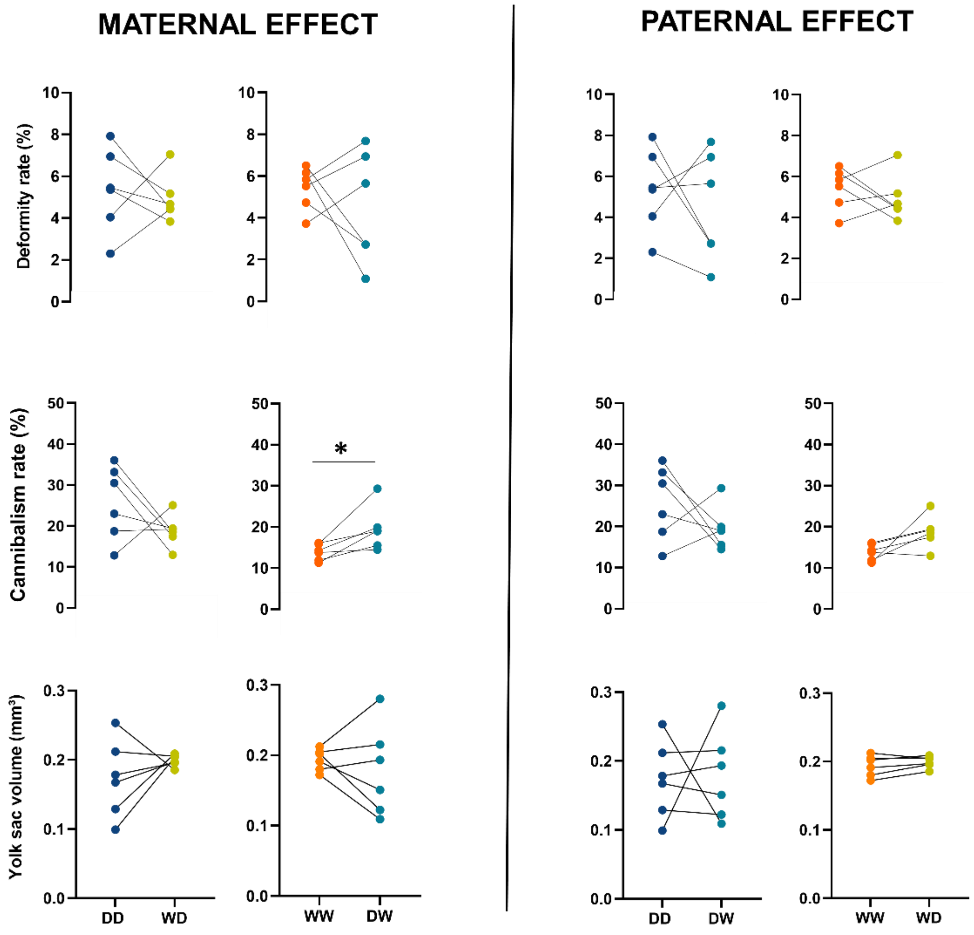

Figure S1: Deformity and Cannibalism rate (%) and yolk sac (mm^3) measured for all the Eurasian perch families. Asterisk show significance difference (*p< 0.05). Additional statistical information is provided in Additional File 6.

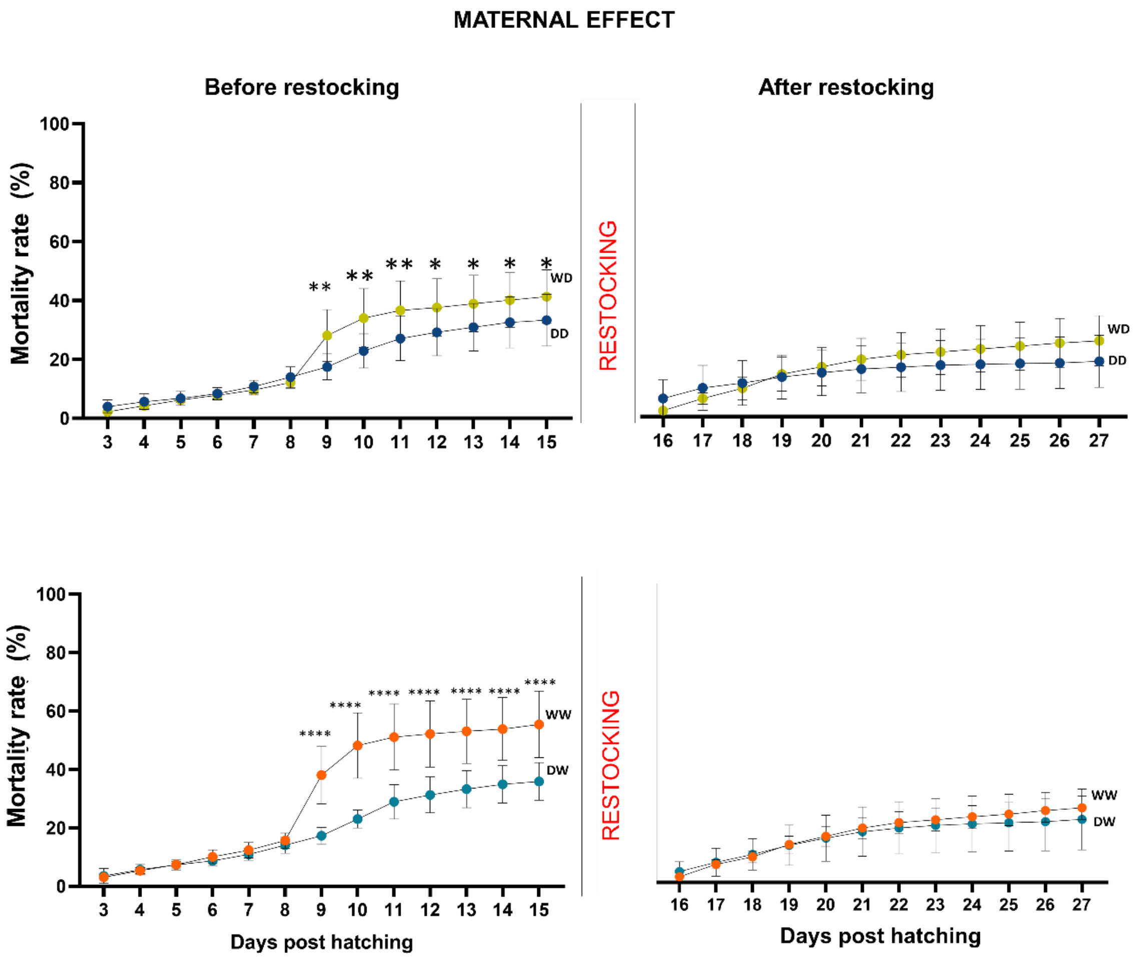

Figure S2: Cumulative mortality (mean ± SD) before and after restocking for all crossings of Eurasian perch larvae analysed for maternal-effect. Error bars indicate standard deviation (SD). Asterisk show significance difference (*p< 0.05, **p < 0.01, ****p < 0.0001). Additional statistical information is provided in Additional File 6.

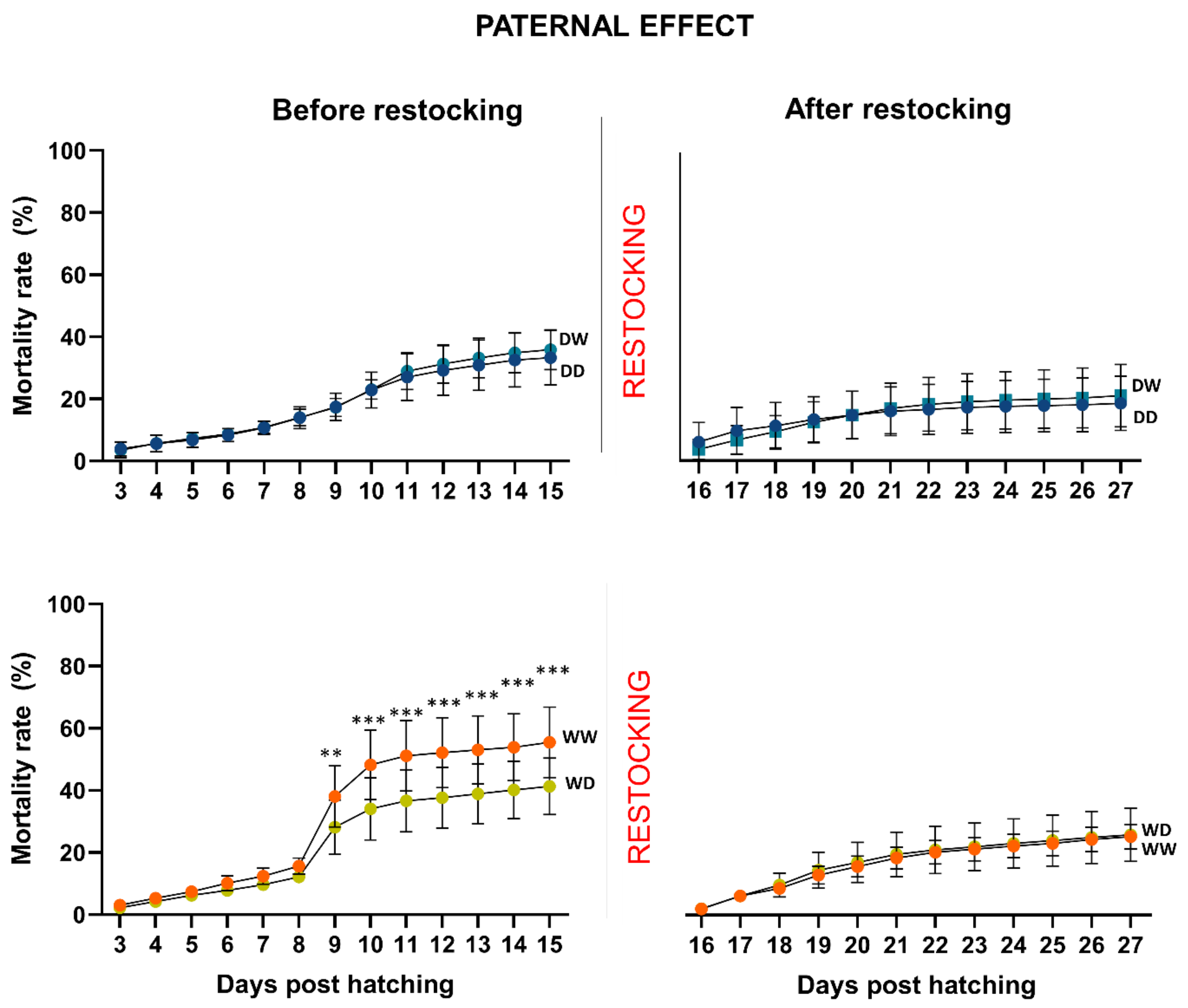

Figure S3: Cumulative mortality (mean ± SD) before and after restocking for all crossings of Eurasian perch larvae analysed for paternal-effect. Error bars indicate standard deviation (SD). Asterisk show significance difference (**p < 0.01, ***p < 0.001). Additional statistical information is provided in Additional File 6.

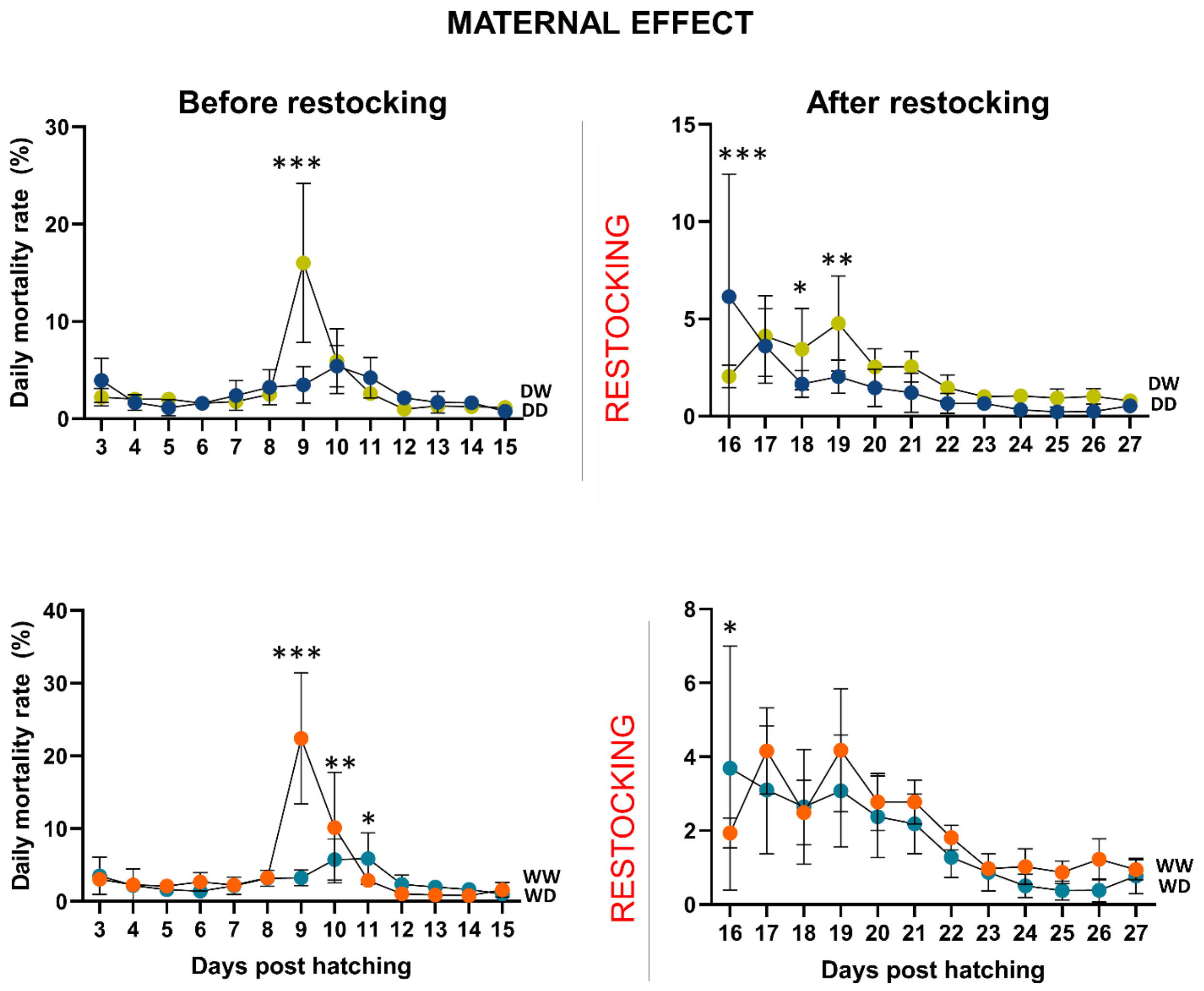

Figure S4: Daily mortality (mean ± SD) before and after restocking for all crossings of Eurasian perch larvae analysed for maternal-effect. Error bars indicate standard deviation (SD). Asterisk show significance difference (*p < 0.05, **p < 0.01, ***p < 0.001). Additional statistical information is provided in Additional File 6.

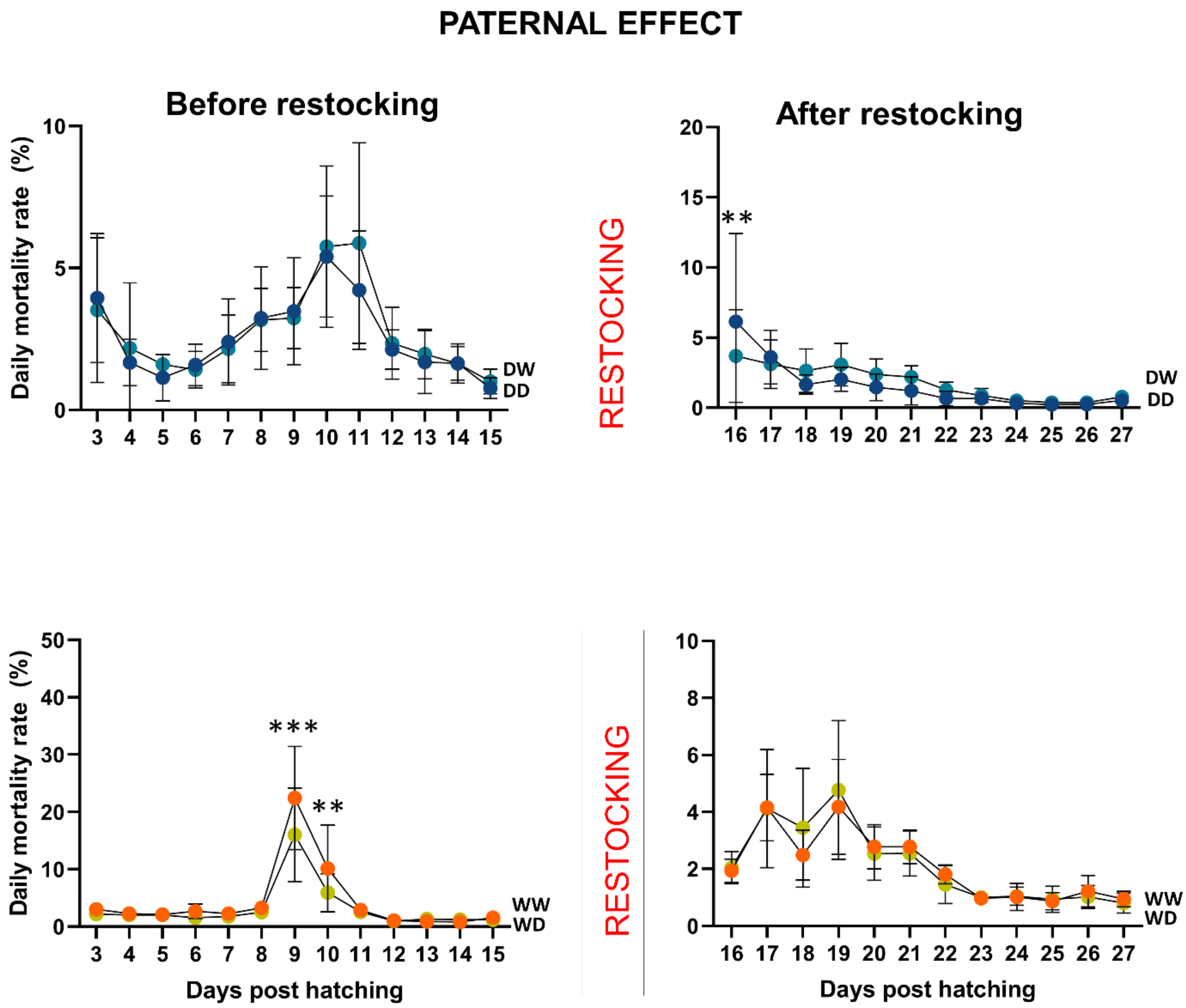

Figure S5: Daily mortality (mean ± SD) before and after restocking for all crossings of Eurasian perch larvae analysed for paternal-effect. Error bars indicate standard deviation (SD). Asterisk show significance difference (** $p < 0.01$, *** $p < 0.001$). Additional statistical information is provided in Additional File 6.

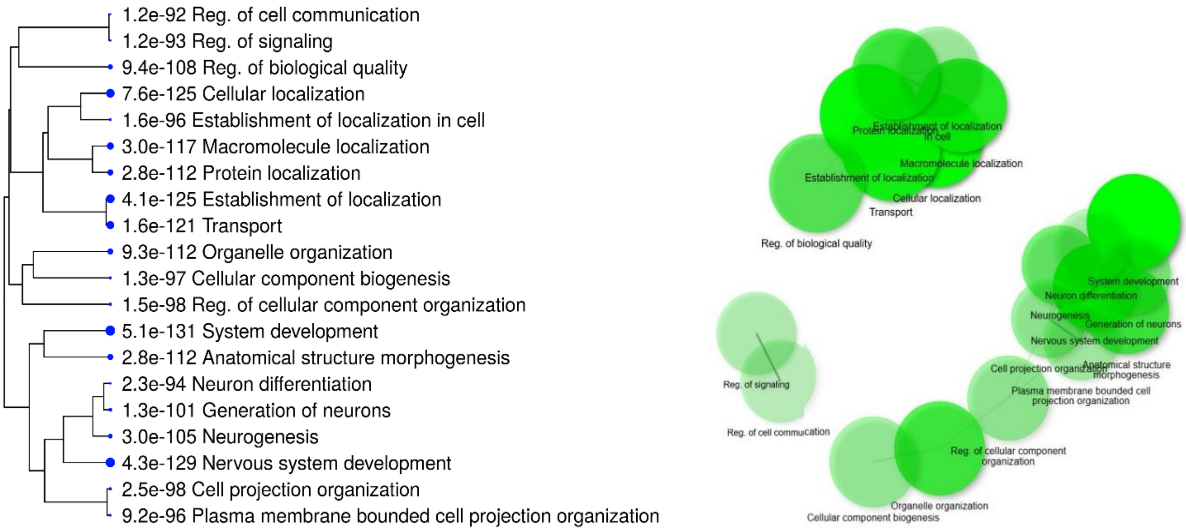

Figure S6: **Tree view and network visualization showing the 20 most significantly enriched GO (biological process)** for non-differentially expressed genes**.**

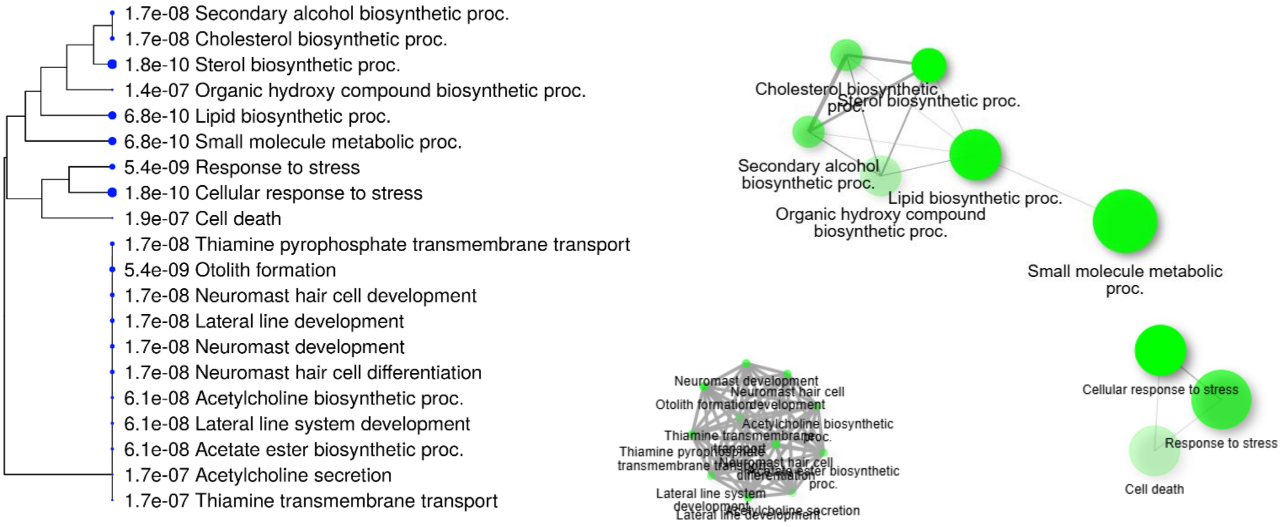

Figure S7: **Tree view and network visualization showing the 20 most significantly enriched GO (biological process)** for purely maternal-effect genes**.**

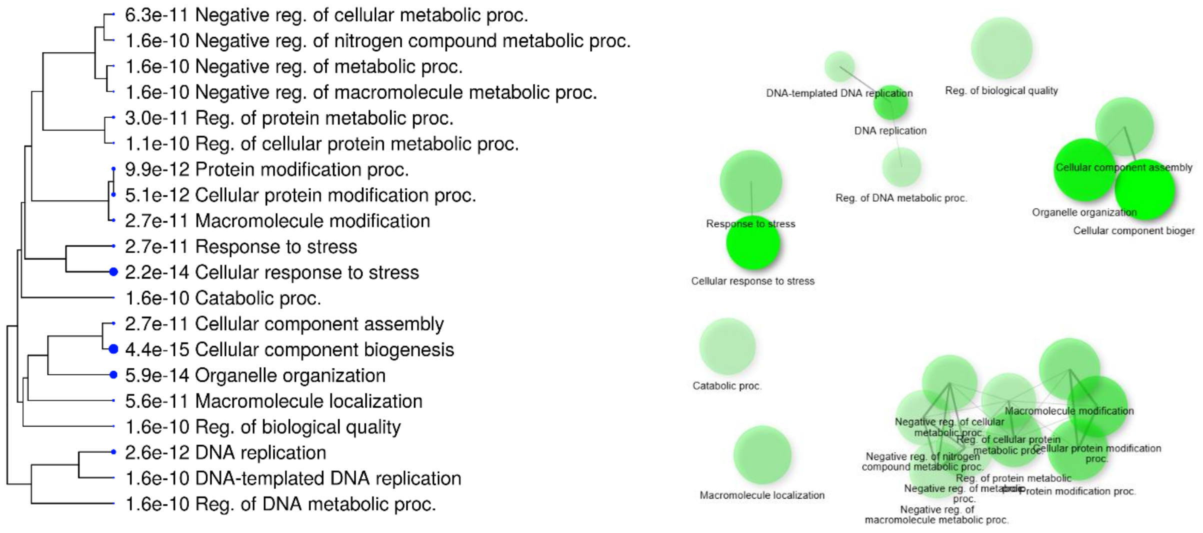

Figure S8: **Tree view and network visualization showing the 20 most significantly enriched GO (biological process)** for all the conditionally maternal-effect genes**.**
