# Supplementary material for: Dynamic interplay of maternal and paternal contributions to offspring phenotype in Eurasian perch
Source: BMC Biol. 2026 Apr 20;24:129. doi: 10.1186/s12915-026-02602-x (PMC13224677; doi:10.1186/s12915-026-02602-x)
Supplement: Supplementary file 3 — Additional file 3: Broodstock characteristics for both wild and domesticated individuals and milt evaluation with CASA after cryopreservation. WBW: Wet Body Weight, LT: Total Length; W: Weight. ALH: Amplitude of Lateral Head Displacement; LIN: Linearity; VAP: Average Path Velocity; VCL: Curvilinear Velocity; VSl: Straight Velocity; MOT: percentage of sperm motility. [file 12915_2026_2602_MOESM3_ESM.pdf]

Additional file 3: Broodstock characteristics for both wild and domesticated individuals and milt evaluation with CASA after cryopreservation. WBW: Wet Body Weight, LT: Total Length; W: Weight. ALH: Amplitude of Lateral Head Displacement; LIN: Linearity; VAP: Average Path Velocity; VCL: Curvilinear Velocity; VSl: Straight Velocity; MOT: percentage of sperm motility.

MALES									
WILD	WBW (g)	LT (cm)	[ ] fresh semen (in billions)	MOT %	LIN %	ALH (μm^{-1})	VAP (μms^{-1})	VCL (μm^{-1})	VSL (μms^{-1})
Male 1	170	23.4	30.9	78.4	80.7	6.0	172.2	187.5	155.9
Male 2	178	24.7	27.5	76.0	77.0	7.5	175.2	192.2	152.8
Male 3	335	31.2	27.7	79.6	75.1	8.3	188.0	203.5	162.3
Male 4	324	31.1	27.9	79.9	73.9	8.1	175.4	190.5	147.6
Male 5	190	25.2	21.2	77.3	72.4	9.9	187.2	204.0	151.9
Male 6	129	22.8	32.8	80.5	73.5	10.4	205.1	223.5	169.7
DOM									
Male 1	666	35.5	26.4	82.4	75.9	5.7	134.1	157.9	123.2
Male 2	467	33.7	25.9	69.6	68.5	4.8	105.9	137.0	97.2
Male 3	468	34.0	34.2	73.9	72.1	4.8	115.8	144.5	105.6
Male 4	450	33.5	29.2	87.0	75.0	5.7	134.1	160.2	121.9
Male 5	615	35.8	28.3	73.5	68.2	5.0	116.2	145.1	105.1
Male 6	491	32.9	37.5	85.6	56.4	5.3	78.4	120.6	66.8

FEMALES				
WILD	WBW(g)	LT (cms)	W ribbon (g)	Egg density (g^{-1})
Female 1	470	28.7	110	545
Female 2	603	32.8	124	517
Female 3	409	30	84	557
Female 4	438	33.7	130	518
Female 5	293	28	75	504
Female 6	394	30	84	463
DOM				
Female 1	369	31.1	92	404
Female 2	549	30.6	142	479
Female 3	563	33.5	159	485
Female 4	427	30.9	106	552
Female 5	447	32.2	150	601
Female 6	539	33.7	88	514
