# Supplementary material for: Dynamic interplay of maternal and paternal contributions to offspring phenotype in Eurasian perch
Source: BMC Biol. 2026 Apr 20;24:129. doi: 10.1186/s12915-026-02602-x (PMC13224677; doi:10.1186/s12915-026-02602-x)
Supplement: Supplementary file 5 — Additional file 5: List of the primers used for RT-qPCR in the study. * = housekeeping genes. [file 12915_2026_2602_MOESM5_ESM.pdf]

Supplementary file S4: Details of the primers used for qRT PCR in the study. *=housekeeping genes

Gene name	Sequences	Amplicon length (bp)
*beta actin [XM_039824642.1]	F: ACCTTCTACAACGAGCTGAGAGT	153
	R: AGTGGTACGACCAGAGGCATA	
*rpl8 [XM_039817090.1]	F: CGTGACCCATACCGCTTCAA	110
	R: CTCTTCCACGCAGCAGATGA	
*idh3b [XM_039797081.1]	F: TCATGTGAATAGCCTGCCGG	130
	R: TTCGATCACACCCGTCACAC	
*atp5f1c [XM_039792329.1]	F: AAGCGCCAGAAGACAAGTCA	181
	R: TGCAGCAGGTTTCTCAGCTT	
*naca [XM_039799779.1]	F: GCCAAGAATGTGCAGGGTTC	123
	R: GGGGGTAGTGGATTTTGGAGT	
*atp5pb [XM_039800828.1]	F: CCAGGCGGTACAATGAAGAT	128
	R: GCGGAGCAATGTGGTAATTT	
crtac1a [XM_039784574.1]	F: CGTCGTGGTGTCGCTAATCA	120
	R: CTCTAAGAGGGCGTGGGGAC	
slc16a7 [XM_039799069.1]	F: TACGGGCTTTGTGGACATGT	204
	R: GCGAAAACCATGCCGAAACT	
kdr [XM_039806878.1]	F: GTACACTTGCATAGCCACGC	131
	R: CCGAGGTCATCAACACGAGA	
cox5b [XM_039783533.1]	F: GGCCCTCAAACATGGAAAGG	122
	R: CACAAAGACAGCCAACCAGC	

lsp1a [XM_039809086.1]	F: GCCTTCAGTCCCTTCAGTCC	135
	R: GTCGTCTATCTTGGGCAGCA	
bin2b [XM_039797340.1]	F: CAAAGTCAGAGGCGGGAAGT	152
	R: GTCTCCTCCTGCACCTTAGC	
